# Non-communicable disease, sociodemographic factors, and risk of death from infection: a UK Biobank observational cohort study

**DOI:** 10.1016/S1473-3099(20)30978-6

**Published:** 2021-08

**Authors:** Michael Drozd, Mar Pujades-Rodriguez, Patrick J Lillie, Sam Straw, Ann W Morgan, Mark T Kearney, Klaus K Witte, Richard M Cubbon

**Affiliations:** a Leeds Institute of Cardiovascular and Metabolic Medicine, School of Medicine, University of Leeds, Leeds, UK; b Leeds Institute of Health Sciences, School of Medicine, University of Leeds, Leeds, UK; c IQVIA, London, UK; d Department of Infection, Castle Hill Hospital, Hull University Hospitals NHS Trust, Kingston Upon Hull, UK; e NIHR Leeds Biomedical Research Centre, Leeds Teaching Hospitals NHS Trust, Leeds, UK

## Abstract

**Background:**

Non-communicable diseases (NCDs) have been highlighted as important risk factors for COVID-19 mortality. However, insufficient data exist on the wider context of infectious diseases in people with NCDs. We aimed to investigate the association between NCDs and the risk of death from any infection before the COVID-19 pandemic (up to Dec 31, 2019).

**Methods:**

For this observational study, we used data from the UK Biobank observational cohort study to explore factors associated with infection death. We excluded participants if data were missing for comorbidities, body-mass index, smoking status, ethnicity, and socioeconomic deprivation, and if they were lost to follow-up or withdrew consent. Deaths were censored up to Dec 31, 2019. We used Poisson regression models including NCDs present at recruitment to the UK Biobank (obesity [defined by use of body-mass index] and self-reported hypertension, chronic heart disease, chronic respiratory disease, diabetes, cancer, chronic liver disease, chronic kidney disease, previous stroke or transient ischaemic attack, other neurological disease, psychiatric disorder, and chronic inflammatory and autoimmune rheumatological disease), age, sex, ethnicity, smoking status, and socioeconomic deprivation. Separate models were constructed with individual NCDs replaced by the total number of prevalent NCDs to define associations with multimorbidity. All analyses were repeated with non-infection-related death as an alternate outcome measure to establish differential associations of infection death and non-infection death. Associations are reported as incidence rate ratios (IRR) accompanied by 95% CIs.

**Findings:**

After exclusion of 9210 (1·8%) of the 502 505 participants in the UK Biobank cohort, our study sample comprised 493 295 individuals. During 5 273 731 person-years of follow-up (median 10·9 years [IQR 10·1–11·6] per participant), 27 729 deaths occurred, of which 1385 (5%) were related to infection. Advancing age, male sex, smoking, socioeconomic deprivation, and all studied NCDs were independently associated with the rate of both infection death and non-infection death. Compared with White ethnicity, a pooled Black, Asian, and minority ethnicity group was associated with a reduced risk of infection death (IRR 0·64, 95% CI 0·46–0·87) and non-infection death (0·80, 0·75–0·86). Stronger associations with infection death than with non-infection death were observed for advancing age (age 65 years *vs* 45 years: 7·59, 95% CI 5·92–9·73, for infection death *vs* 5·21, 4·97–5·48, for non-infection death), current smoking (*vs* never smoking: 3·69, 3·19–4·26, *vs* 2·52, 2·44–2·61), socioeconomic deprivation (most *vs* least deprived quintile: 2·13, 1·78–2·56, *vs* 1·38, 1·33–1·43), class 3 obesity (*vs* non-obese: 2·21, 1·74–2·82, *vs* 1·55, 1·44–1·66), hypertension (1·36, 1·22–1·53, *vs* 1·15, 1·12–1·18), respiratory disease (2·21, 1·96–2·50, *vs* 1·28, 1·24–1·32), chronic kidney disease (5·04, 4·28–7·31, *vs* 2·50, 2·20–2·84), psychiatric disease (1·56, 1·30–1·86, *vs* 1·23, 1·18–1·29), and chronic inflammatory and autoimmune rheumatological disease (2·45, 1·99–3·02, *vs* 1·41, 1·32–1·51). Accrual of multimorbidity was also more strongly associated with risk of infection death (five or more comorbidities *vs* none: 9·53, 6·97–13·03) than of non-infection death (5·26, 4·84–5·72).

**Interpretation:**

Several NCDs are associated with an increased risk of infection death, suggesting that some of the reported associations with COVID-19 mortality might be non-specific. Only a subset of NCDs, together with the accrual of multimorbidity, advancing age, smoking, and socioeconomic deprivation, were associated with a greater IRR for infection death than for other causes of death. Further research is needed to define why these risk factors are more strongly associated with infection death, so that more effective preventive strategies can be targeted to high-risk groups.

**Funding:**

British Heart Foundation.

## Introduction

Non-communicable diseases (NCDs) have been highlighted as important risk factors for fatal SARS-CoV-2 infection.^1^, ^2^ With use of the OpenSAFELY primary care health analytics platform based in England, increased risk of COVID-19 death has been independently associated with advancing age, male sex, non-White ethnicity, socioeconomic deprivation, and many NCDs, including the following: severe obesity; diabetes; recently diagnosed cancer; chronic respiratory, cardiac, kidney, and liver disease; neurological disorders, including stroke and dementia; and rheumatological disorders.^1^ Similar conclusions were reached by Docherty and colleagues in an analysis of people hospitalised due to COVID-19 in England and Wales,^3^ specifically finding that death was associated with advancing age, male sex, chronic respiratory, cardiac, kidney, and liver disease, neurological disease, cancer, and obesity (but not diabetes). These data have prompted hypotheses that SARS-CoV-2 specifically interacts with some comorbidities.^4^, ^5^, ^6^ However, the wider context of infectious diseases in people with NCDs is often neglected by such hypotheses, despite infection with pathogens other than SARS-CoV-2 being much more common and potentially offering insight into the conserved and the disease-specific risks of COVID-19.

**Research in context**

**Evidence before this study**

We searched PubMed on June 30, 2020, for original research articles in English using the search terms “(infection[title] OR infectious[title] OR sepsis[title]) AND (death OR mortality) AND (“non-communicable disease” OR comorbidity OR multimorbidity) AND (population OR cohort)”. Our search resulted in 275 relevant studies identified. These revealed that infection contributes to approximately one in five deaths globally and that infection death often occurs in people with non-communicable diseases (NCDs). Many studies have identified NCDs as risk factors for death from specific pathogens, often in narrowly defined cohort or case-control studies. During 2020, large cohort studies have identified many NCDs as independent risk factors for fatal SARS-CoV-2 infection, but comparable analyses of death from any infection have not been done.

**Added value of this study**

The independent risks posed by specific NCDs (or multimorbidity) for death from any acute infection have not previously been explored in observational cohort studies. We found that although all studied NCDs were associated with greater risk of infection death, only severe obesity, hypertension, respiratory disease, chronic kidney disease, psychiatric disease, and chronic inflammatory and autoimmune rheumatological disease were associated with a greater risk of infection death than of non-infection death. The presence of multimorbidity (even with just two comorbid illnesses) was also associated with a greater risk of infection death than of non-infection death, as were advancing age, current smoking, and socioeconomic deprivation. Notably, the factors we found to be associated with infection death in the pre-COVID-19 era were similar to those identified in studies focused on COVID-19 mortality, with the exception of Black, Asian, and minority ethnicity, which was associated with lower risk of infection death than White ethnicity.

**Implications of all the available evidence**

People with some NCDs (together with multimorbidity, smoking, and socioeconomic deprivation) are at particularly increased risk of infection death, and strategies to discern and address the underlying mechanisms should be developed. Ongoing work addressing NCDs and sociodemographic factors as risk factors for fatal COVID-19 should also consider the wider context of how these factors are associated with death from any other infection and death unrelated to infection.

NCDs can be the sequelae of infectious diseases—for example, as a result of carcinogenesis or chronic organ dysfunction—but are also important risk factors for fatal acute infection—for example, as a result of sepsis or acute organ dysfunction. The Global Burden of Disease Study revealed that 11 million sepsis-related deaths occurred during 2017, amounting to almost 20% of all deaths.^7^ Sepsis-related death was more common in men, older individuals (and infants), and socioeconomically deprived populations. Moreover, about 40% of cases had an NCD listed as the underlying cause of death, with cardiovascular diseases, diabetes, liver disease, chronic obstructive pulmonary disease, and kidney disease all being commonly implicated. Hence, clear parallels can be seen between the risk factors for sepsis death and COVID-19 death.

Despite a 30% reduction in global sepsis-related deaths between 1990 and 2017,^7^ the issue of life-threatening infection in people with NCDs is likely to become more important in the context of globally increasing rates of multimorbidity and antimicrobial resistance.^8^, ^9^ Moreover, as populations become more affluent, NCDs are proportionally more common as an underlying cause of sepsis death,^7^ underlining the growing relevance to many economically developing populations. Despite the scale and impact of this issue, little prospective research is published about the independent and additive risks posed by specific NCDs, and multimorbidity, for acute infection-related death. To address this gap, we investigated the association between NCDs and the risk of death from any infection by analysing the well characterised UK Biobank cohort study using follow-up data to the end of 2019 (before the COVID-19 pandemic).

## Methods

### Study design and data collection

For this observational study, we used data from the UK Biobank cohort, a population-based prospective study that consists of 502 505 people aged 37–73 years. The resource was developed with use of funding from the UK Government and biomedical research charities to improve understanding of disease, and it is an open access resource for all researchers. Full details of the study design and conduct are available at the UK Biobank website. Participants were recruited between 2006 and 2010, and attended one of 22 assessment centres across England, Scotland, and Wales; although the cohort is not representative of the whole UK population regarding socioeconomic deprivation, some NCDs, and ethnic minorities, it allows the assessment of exposure–disease relationships.^10^ Baseline biological measurements were recorded, and participants completed a touchscreen and nurse-led questionnaire, as described elsewhere.^11^ UK Biobank received ethical approval from the National Health Service (NHS) Research Ethics Service (11/NW/0382), and we did this analysis under application number 59585. All participants provided written informed consent. There was no patient or public involvement in the planning of this analysis and no prespecified analysis plan is published.

### Assessment of demographic factors and morbidity

Age, sex, ethnicity, and socioeconomic status were defined at recruitment to UK Biobank and were considered as potential risk factors for death. Ethnicity was participant-classified within UK Biobank-defined categories of White, Mixed, Asian or British Asian, Black or British Black, Chinese, or other ethnic groups; due to the small number of people (and deaths) in each non-White ethnic group, these were pooled into a Black, Asian, and minority ethnic (BAME) group in most analyses, although they were considered separately in a sensitivity analysis. Smoking status was self-reported as never, former, or current at the point of recruitment. Socioeconomic deprivation was measured with the Townsend score and categorised into quintiles. Obesity was classified using WHO's categorisation according to body-mass index: class 1 (30·0–34·9 kg/m^2^), class 2 (35·0–39·9 kg/m^2^), and class 3 (≥40 kg/m^2^). Self-reported medical conditions recorded solely at study recruitment during face-to-face interview with a nurse were used to classify morbidities into groups (appendix pp 1–2). We used clinical consensus before our analyses to select a range of morbidities representing a broad spectrum of common disease groups, including the following: hypertension, chronic heart disease (ischaemic heart disease and heart failure), chronic respiratory disease, diabetes, previous cancer, chronic liver disease, chronic kidney disease, previous stroke or transient ischaemic attack, other neurological disease, psychiatric disease, and chronic inflammatory and autoimmune rheumatic disease.^12^ The number of comorbidities was calculated for each participant. Participants were excluded from the analysis if they had missing data for comorbidities. Participants were also excluded if they were lost to follow-up or withdrew consent.

### Mortality ascertainment

Mortality information was provided by the UK Biobank data portal, which uses linked national death registry data from NHS Digital for participants in England and Wales and from the NHS Central Register, part of the National Records of Scotland, for participants in Scotland. In our analysis, we censored deaths up until Dec 31, 2019, to ensure included deaths occurred before the first reported case of COVID-19 in the UK.^13^ Using codes from the International Classification of Diseases (tenth revision), we classified the main cause of death as infection related or non-infection related (appendix pp 3, 17–43).

### Statistical analysis

Continuous variables are presented as mean (SD) or median (IQR) if non-normally distributed, and categorical variables are presented as number (%). We plotted Kaplan-Meier curves to illustrate mortality rates. Absolute rates of death observed in specific categories of participants were defined by dividing the number of observed deaths in each category by the respective duration of follow-up, and expressed per 1000 person-years. We estimated adjusted cause-specific mortality incidence rate ratios (IRRs) using Poisson regression models; exposure time was modelled, but time-varying covariates were not used, and the calendar year of recruitment was not included in models because of the narrow recruitment period. Models were adjusted for all covariates, including age, sex, socioeconomic deprivation quintile, smoking status, obesity, hypertension, chronic heart disease, chronic respiratory disease, diabetes, cancer (previous or current), chronic liver disease, chronic kidney disease, previous stroke or transient ischaemic attack, other neurological disease, psychiatric disorder, and rheumatological disease. We used correlation matrices of Poisson model's coefficients to assess correlations between covariates; no correlation coefficients higher than 0·3 or lower than –0·3 were observed. To assess the association of multimorbidity with infection death, irrespective of the specific morbidities, we also separately modelled the number of comorbidities (up to a maximum of five, because few participants had six or more, limiting confidence in estimates) in place of the individual comorbidity variables. We modelled age using restricted cubic splines with five knots for infection death analyses and four knots for non-infection death analyses, because these provided the best fit as assessed by the Akaike information and the Bayesian criterion (models including categorical, linear, or cubic splines with three, four, and five knots and first-degree and second-degree fractional polynomials were compared). Therefore, presented IRR estimates for age pertain to specific age points (50, 55, 60, and 65 years, compared with 45 years), not an age category (appendix p 15).

We did several sensitivity analyses. First, we assessed unadjusted models; models adjusted for age and sex; models adjusted for age, sex, and demographic factors; and fully adjusted models. Second, we assessed Mixed, Asian or British Asian, Black or British Black, Chinese, and other ethnicity as individual groups, rather than pooling them into a single BAME group. Third, we compared risk of death (infection and non-infection) by lower respiratory tract infection versus other infection. Fourth, we excluded deaths occurring after 5 years or 9 years of recruitment, which might have been caused by NCDs diagnosed after recruitment to the UK Biobank cohort. And fifth, we assessed age-adjusted and sex-adjusted models in the study cohort (excluding individuals from the UK Biobank cohort with missing data) versus the full UK Biobank cohort. We used quantitative bias analysis using e-values^14^ to define the degree of unmeasured confounding that would be required to nullify observed associations between socioeconomic deprivation and infection death. All tests were two-sided and statistical significance was defined as p<0·05. All statistical analyses were done with Stata/MP (version 16.1).

### Role of the funding source

The funder of the study had no role in study design, data collection, data analysis, data interpretation, or writing of the report.

## Results

**Figure 1:**
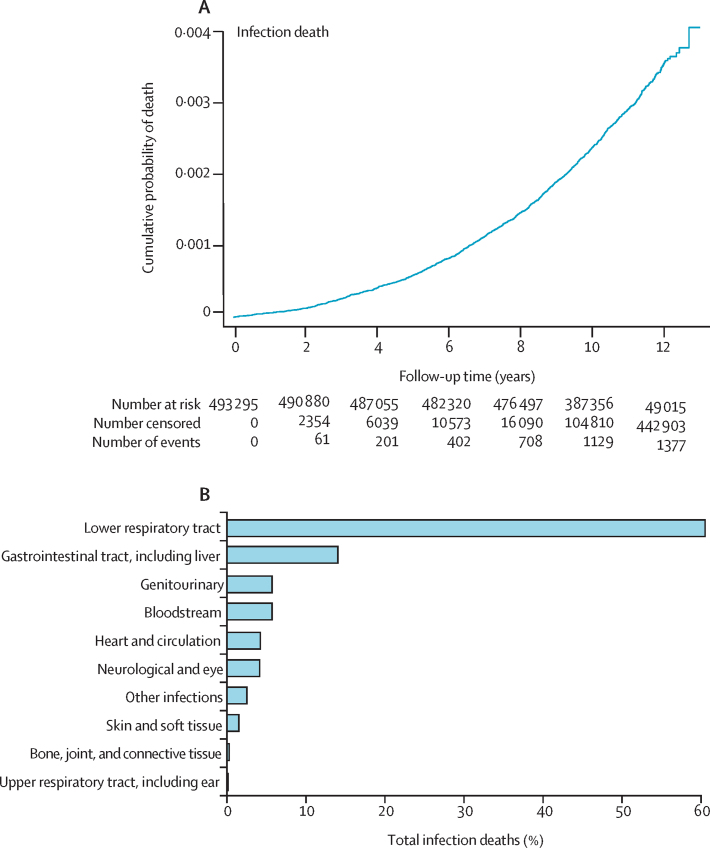
Timing and classification of infection deaths (A) Kaplan-Meier mortality curve of cumulative infection deaths during follow-up. (B) Classification of infection deaths.

Among the UK Biobank cohort of 502 505 people, we excluded 9210 (1·8%) because of missing baseline data or long-term follow-up data. These included exclusions due to missing data for comorbidities (863 individuals), body-mass index (3106 individuals), smoking status (2949 individuals), ethnicity (2777 individuals), and socioeconomic deprivation quintile (624 individuals), and individuals lost to follow-up or who withdrew consent (1298 individuals). These exclusions resulted in a study sample of 493 295 people. In the study cohort, 1385 infection deaths occurred before Dec 31, 2019, during 5 273 731 person-years of follow-up (median 10·9 years [IQR 10·1–11·6] per participant), accounting for 5% of the total 27 729 deaths (figure 1A, appendix p 16). The most common fatal infections involved the lower respiratory tract (840 events [60·6%]), gastrointestinal tract (196 events [14·2%]), and genitourinary tract (82 events [5·9%]); data for all causes are presented in figure 1B and the appendix (p 4). Compared with survivors, people who died with infection were older, more often men, more socioeconomically deprived, more commonly current or former smokers, and more likely to have a wide range of NCDs (table 1). Broadly similar differences were observed when comparing survivors with people who died from causes other than infection, although cancer was markedly more common in people who died from non-infection causes than in people who died from infection. Absolute rates of infection and non-infection death per 1000 person-years are shown in table 2.

**Table 1:** Participant characteristics at study recruitment

		**Survivors (n=465 566)**	**Infection death (n=1385)**	**Non-infection death (n=26 344)**
Age, years				
	<45	50 081 (10·8%)	23 (1·7%)	599 (2·3%)
	45 to <50	63 617 (13·7%)	40 (2·9%)	1205 (4·6%)
	50 to <55	72 683 (15·6%)	103 (7·4%)	2161 (8·2%)
	55 to <60	85 185 (18·3%)	195 (14·1%)	3881 (14·7%)
	60 to <65	111 064 (23·9%)	383 (27·7%)	7924 (30·1%)
	≥65	82 936 (17·8%)	641 (46·3%)	10 574 (40·1%)
Sex				
	Men	207 883 (44·7%)	842 (60·8%)	15 712 (59·6%)
	Women	257 683 (55·3%)	543 (39·2%)	10 632 (40·4%)
Ethnicity				
	White	440 402 (94·6%)	1344 (97·0%)	25 453 (96·6%)
	Mixed	2797 (0·6%)	4 (0·3%)	104 (0·4%)
	Asian	9106 (2·0%)	19 (1·4%)	338 (1·3%)
	Black	7534 (1·6%)	12 (0·9%)	249 (0·9%)
	Chinese	1502 (0·3%)	1 (0·1%)	37 (0·1%)
	Other	4225 (0·9%)	5 (0·4%)	163 (0·6%)
SED quintile				
	1 (least deprived)	93 973 (20·2%)	170 (12·3%)	4555 (17·3%)
	2	93 776 (20·1%)	206 (14·9%)	4640 (17·6%)
	3	93 483 (20·1%)	240 (17·3%)	4936 (18·7%)
	4	93 112 (20·0%)	295 (21·3%)	5254 (19·9%)
	5 (most deprived)	91 222 (19·6%)	474 (34·2%)	6959 (26·4%)
Smoking				
	Never	259 721 (55·8%)	439 (31·7%)	10 072 (38·2%)
	Former	159 304 (34·2%)	582 (42·0%)	11 128 (42·2%)
	Current	46 541 (10·0%)	364 (26·3%)	5144 (19·5%)
Obesity				
	Not obese	352 374 (75·7%)	928 (67·0%)	18 206 (69·1%)
	Class 1	81 466 (17·5%)	281 (20·3%)	5471 (20·8%)
	Class 2	22 992 (4·9%)	96 (6·9%)	1828 (6·9%)
	Class 3	8734 (1·9%)	80 (5·8%)	839 (3·2%)
Hypertension		119 429 (25·7%)	667 (48·2%)	10 701 (40·6%)
Chronic cardiac disease		19 671 (4·2%)	250 (18·1%)	3546 (13·5%)
Chronic respiratory disease		59 068 (12·7%)	385 (27·8%)	4451 (16·9%)
Diabetes		21 206 (4·6%)	228 (16·5%)	3317 (12·6%)
Cancer		35 818 (7·7%)	177 (12·8%)	5012 (19%)
Chronic liver disease		797 (0·2%)	12 (0·9%)	147 (0·6%)
Chronic kidney disease		1001 (0·2%)	29 (2·1%)	241 (0·9%)
Previous stroke or TIA		7214 (1·5%)	104 (7·5%)	1274 (4·8%)
Other neurological disease		5726 (1·2%)	57 (4·1%)	763 (2·9%)
Psychiatric disorder		27 578 (5·9%)	141 (10·2%)	1930 (7·3%)
Rheumatological disease		10 023 (2·2%)	97 (7·0%)	972 (3·7%)

Data are n (%). BMI=body-mass index. SED=socioeconomic deprivation. TIA=transient ischaemic attack.

**Table 2:** Absolute unadjusted rates of infection death and non-infection death per 1000 person-years of follow-up according to baseline characteristics

		**Infection death**	**Non-infection death**
Age, years			
	<45	0·04 (0·03–0·06)	1·08 (1·00–1·17)
	45 to <50	0·06 (0·04–0·08)	1·71 (1·61–1·81)
	50 to <55	0·13 (0·10–0·15)	2·66 (2·55–2·78)
	55 to <60	0·20 (0·18–0·23)	4·04 (3·91–4·17)
	60 to <65	0·30 (0·27–0·33)	6·26 (6·13–6·40)
	≥65	0·66 (0·61–0·71)	10·81 (10·61–11·01)
Sex			
	Men	0·35 (0·33–0·38)	6·60 (6·49–6·70)
	Women	0·19 (0·17–0·20)	3·68 (3·61–3·75)
Ethnicity			
	White	0·27 (0·25–0·28)	5·09 (5·03–5·15)
	BAME	0·15 (0·11–0·20)	3·27 (3·06–3·49)
SED quintile			
	1	0·16 (0·14–0·19)	4·27 (4·15–4·40)
	2	0·19 (0·17–0·22)	4·38 (4·26–4·51)
	3	0·23 (0·20–0·26)	4·67 (4·54–4·80)
	4	0·28 (0·25–0·31)	5·00 (4·87–5·14)
	5	0·45 (0·42–0·50)	6·68 (6·52–6·84)
Smoking			
	Never	0·15 (0·14–0·17)	3·46 (3·40–3·53)
	Former	0·32 (0·30–0·35)	6·12 (6·01–6·24)
	Current	0·66 (0·60–0·74)	9·40 (9·14–9·66)
Obesity			
	Non-obese	0·23 (0·22–0·25)	4·58 (4·51–4·64)
	Class 1	0·30 (0·27–0·34)	5·89 (5·74–6·05)
	Class 2	0·36 (0·30–0·44)	6·92 (6·61–7·24)
	Class 3	0·79 (0·63–0·98)	8·24 (7·70–8·82)
Hypertension		0·48 (0·45–0·52)	7·75 (7·60–7·89)
Chronic cardiac disease		1·04 (0·92–1·18)	14·77 (14·29–15·26)
Chronic respiratory disease		0·57 (0·51–0·63)	6·56 (6·37–6·76)
Diabetes		0·90 (0·79–1·02)	13·06 (12·62–13·51)
Cancer		0·42 (0·36–0·49)	11·95 (11·62–12·28)
Chronic liver disease		1·23 (0·70–2·16)	15·01 (12·77–17·64)
Chronic kidney disease		2·33 (1·62–3·35)	19·33 (17·04–21·93)
Previous stroke or TIA		1·18 (0·98–1·43)	14·49 (13·71–15·30)
Other neurological disease		0·83 (0·64–1·08)	11·17 (10·41–12·00)
Psychiatric disorder		0·45 (0·38–0·53)	6·13 (5·86–6·41)
Rheumatological disease		0·83 (0·68–1·01)	8·28 (7·78–8·82)

Data are rates per 1000 person-years (95% CI). BAME=Black, Asian, and minority ethnicity. BMI=body-mass index. SED=socioeconomic deprivation. TIA=transient ischaemic attack.

**Figure 2:**
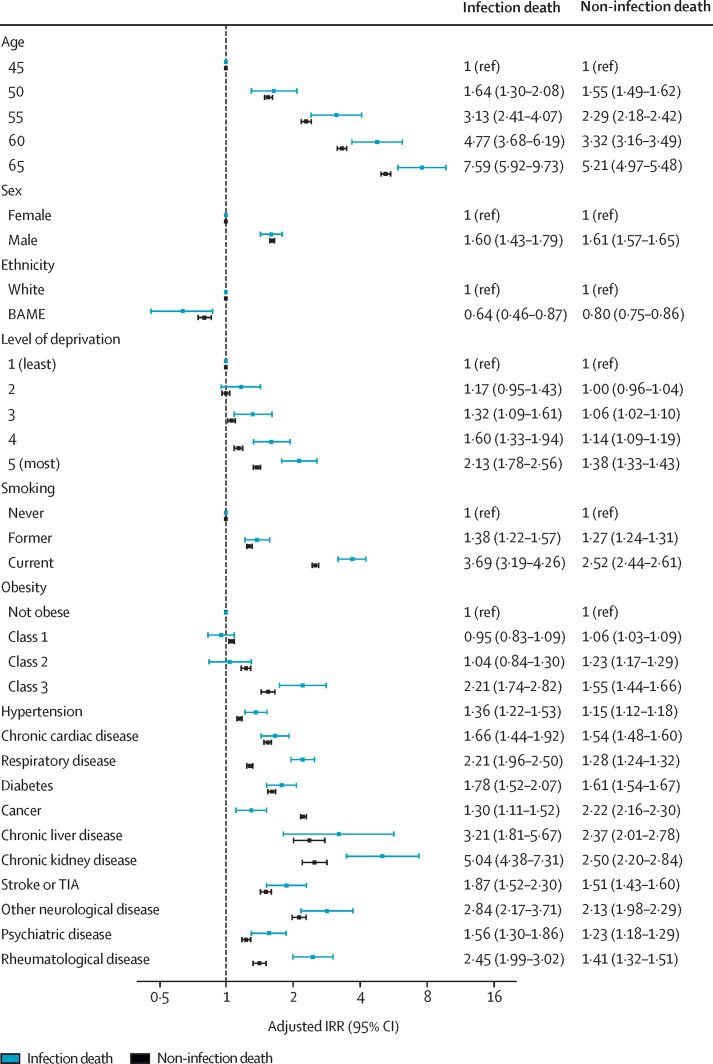
Association between participant characteristics and risk of infection death or non-infection death Adjusted IRRs for infection death or non-infection death obtained from multivariate Poisson regression analysis are shown with 95% CIs. IRR estimates for age pertain to specific age points (age was modelled by use of restricted cubic splines with five knots for infection death analyses and four knots for non-infection death analyses). BAME=Black, Asian, and minority ethnicity. IRR=incidence rate ratio. TIA=transient ischaemic attack.

By assessing IRRs for the association between NCDs and infection death, we found that advancing age, male sex, severe obesity, smoking, socioeconomic deprivation, and all individual NCDs were independently associated with the risk of infection death (figure 2; unadjusted models and models adjusted for age and sex or age, sex, and demographics are shown in appendix pp 5–6). However, BAME was associated with a lower risk of infection death than White ethnicity (IRR 0·64, 95% CI 0·46–0·87), with IRRs remaining below 1 when individual BAME groups were modelled, although with 95% CIs crossing 1 (appendix pp 7–8). Advancing age, male sex, obesity, smoking, socioeconomic deprivation, and all individual NCDs were also independently associated with increased risk of non-infection death; however, BAME was again associated with lower risk (0·80, 0·75–0·86; figure 2). Only the following factors were associated with greater IRRs for infection than for non-infection death: current smoking, socioeconomic deprivation, class 3 obesity, hypertension, chronic respiratory disease, chronic kidney disease, psychiatric disease, and chronic inflammatory and autoimmune rheumatological disease (figure 2). By contrast, cancer was associated with a greater risk of non-infection death than infection death (figure 2).

Because lower respiratory tract infection accounted for the majority of infection deaths in our analysis (figure 1B, appendix p 4), we did a sensitivity analysis evaluating the association between NCDs and risk of death from lower respiratory tract infection or from other infections (appendix p 9). Current smoking and chronic respiratory disease were associated with a greater risk of death from lower respiratory tract infection than from other infection. The large 95% CIs in this analysis preclude robust comparisons, but advancing age, male sex, and increasing socioeconomic deprivation were also associated with nominally larger risks of death from lower respiratory tract infection than from other infection. An additional sensitivity analysis excluding deaths occurring after 5 years or 9 years of follow-up showed associations of similar magnitude between NCDs and infection or non-infection death over time (appendix p 10). Given that 9210 people (56 deaths) from the full UK Biobank cohort were excluded from our analyses because of missing data, we also report results of models adjusted for age and sex done separately for for the full UK Biobank population and for our study population (appendix pp 11–12). These models showed almost identical associations between NCDs and infection or non-infection death, suggesting that the exclusion of 9210 people did not substantially bias our estimates.

**Figure 3:**
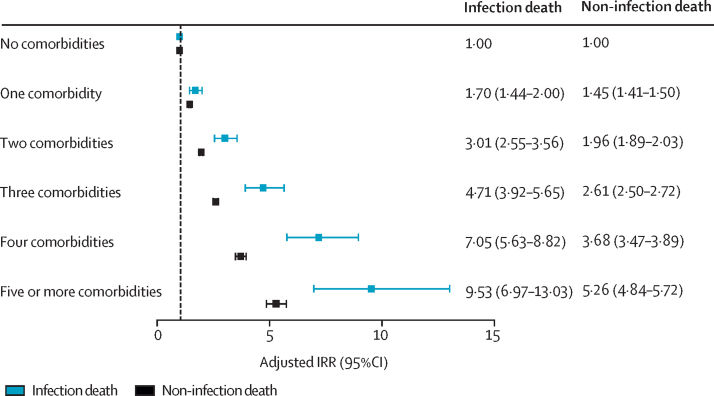
Association between multimorbidity and risk of infection death or non-infection death IRRs for infection death or non-infection death obtained from multivariate Poisson regression analysis, adjusting for age, sex, socioeconomic deprivation, smoking, and ethnicity, are shown with 95% CIs. IRR=incidence rate ratio.

Finally, to explore the effect of multimorbidity, we did alternate analyses including the number of NCDs rather than individual diseases, while also accounting for age, sex, smoking status, ethnicity, and socioeconomic deprivation quintile. This analysis showed increasing risk of infection death as the number of NCDs increased (figure 3; appendix p 13, including unadjusted models and models adjusted for age and sex). Notably, although an increasing risk of non-infection death with increasing multimorbidity was also observed, this increase was less steep than that for infection death.

## Discussion

To our knowledge, this study reports the largest prospective analysis of risk factors for infection death in people recruited in a community setting. Infection death was more common in men, with advancing age, and with increasing socioeconomic deprivation. All studied NCDs were also independently associated with increased risk of infection death, as was the accrual of multimorbidity. Except for ethnicity, risk factors for infection death were similar to those reported for COVID-19 death, with generally similar IRRs for infection and non-infection death observed. However, some factors conferred greater risk of infection death than of non-infection death, including advancing age, smoking status, increasing socioeconomic deprivation, class 3 obesity, hypertension, chronic respiratory disease, chronic kidney disease, psychiatric disease, chronic inflammatory and autoimmune rheumatological disease, and the accrual of multimorbidity. These high-risk populations might benefit from strategies to prevent or more effectively treat infections.

The existing literature supports the importance of infection as a cause of death in populations with many of the NCDs we have studied, including in people with chronic heart failure,^15^, ^16^ diabetes,^17^ chronic kidney disease,^18^ liver disease,^19^ rheumatological disease,^20^ and neurodegenerative disorders.^21^ Our analysis supports these disease-specific data, but it has the advantage of considering both the additive and independent risk of infection death attributable to multiple NCDs in the same population. Notably, although the accumulation of multimorbidity has been linked with increased risks of all-cause, cardiovascular, and cancer death,^12^ our finding that accumulation of multimorbidity was associated with a greater risk of infection death than of non-infection death is novel. Regarding the larger hazard of infection death associated with socioeconomic deprivation, it is notable that socioeconomic interventions have had major roles in addressing past and present infectious diseases.^22^, ^23^ Although residual confounding relating to unmeasured associates of deprivation could explain this association, this would need to be strong to remove the link because an exploratory quantitative bias analysis of the IRR for infection death comparing socioeconomic deprivation quintile 5 versus 1 (2·13, 95% CI 1·78–2·56; appendix p 5) yielded an e-value of 3·68 (95% CI 2·96–4·56).^14^

The COVID-19 pandemic has brought fresh attention to the association between NCDs and infectious diseases. Although some disagreements exist regarding specific risk factors (probably reflecting study design), the COVID-19 literature broadly suggests that older age, male sex, socioeconomic deprivation, and accrual of NCDs increase the risk of adverse outcomes.^1^, ^3^ This broad agreement with our analysis of risk factors for death related to infection in the pre-COVID-19 era suggests common underlying factors and solutions to these problems. One notable difference was that a pooled BAME group was associated with reduced risk of infection death compared with White ethnicity in our adjusted analyses, but it has frequently been identified as a risk factor for adverse COVID-19 outcomes,^1^, ^24^ including in the UK Biobank cohort.^25^ Although the association in our analysis was significant, our data assessing the association of BAME with outcomes come from a reasonably small number of people and events (26 096 people, with 891 non-infection deaths and 41 infection deaths), and the UK Biobank is not representative of UK ethnic minorities.^10^ These limitations can translate into less precise estimates, especially for individual ethnic groups, as illustrated by our sensitivity analysis including individual ethnic groups (appendix pp 7–8), which exhibited non-significantly reduced IRRs for infection death with very broad confidence intervals. However, our data raise the possibility that the adverse COVID-19 outcomes of BAME groups are not explained by a more generalised susceptibility to infection death.

This study has some limitations. First, UK Biobank is not a nationally representative cohort (particularly relating to socioeconomic deprivation, some NCDs, and ethnic minorities),^10^ thus caution should be used when extrapolating data on infection death prevalence. Moreover, although our finding that infections accounted for about 5% of all deaths is broadly in keeping with Global Burden of Disease data for the UK,^7^ a nationally unrepresentative case-mix means that our risk estimates might be less valid or precise when applied to the general population. We should also highlight the challenges of death certification data, which do not always encompass the complex factors contributing to death. Similarly, the classification of NCD status in the UK Biobank often relies on participant-reported data and was available only at the point of recruitment (as was smoking and deprivation data), so it might not have the fidelity of other approaches and could result in underestimation of the magnitude of associations studied. Although self-reported diabetes data in the UK Biobank have shown reasonable agreement with care records,^26^ we should note that this concordance has not been studied for most NCDs and the proportion of people with NCDs is lower in UK Biobank than in the age-matched and sex-matched UK population (appendix p 14).^10^ Moreover, the classification of most NCDs as being either present or absent neglects the heterogeneity that exists within NCDs, which might have an important influence on risk of infection death. Such misclassification of exposures (and outcome measures) might lead to underestimation of risk estimates. Regarding potentially important missing variables, we were unable to consider the number, nature, or volume of person-to-person contacts, which is particularly relevant to infectious diseases; and we were also unable to comment on the nature of pathogens (eg, bacterial or viral) because the causative pathogen is often not clinically ascertained or recorded. Finally, as with any observational study, it is important not to draw causal inferences from our data, although our findings raise many important and testable hypotheses.

Ongoing work is needed to understand the relative importance of NCDs causing recurrent infections versus increasing the risk of death during any single episode to the overall risk of infection death. Future research should also define mechanisms linking NCDs (and their treatment) to infection death—for example, through altered immune responses to vaccination and infection, inflammatory responses to pathogens, and physiological responses during infection or sepsis. Addressing these uncertainties will help to inform the selection of therapeutic strategies for clinical trials. These could include specific primary and secondary infection prevention measures; improved access to care, monitoring for early detection of infection, and initiation of appropriate antimicrobial treatment; and improved use of high-dependency care. Our data emphasise that research addressing the interaction between infections and NCDs will be important even in a post-COVID-19 era.

## Data sharing

The UK Biobank resource is open to all researchers (https://www.ukbiobank.ac.uk).
