# Supplementary material for: Non-communicable disease, sociodemographic factors, and risk of death from infection: a UK Biobank observational cohort study
Source: Lancet Infect Dis. 2021 Aug;21(8):1184–91. doi: 10.1016/S1473-3099(20)30978-6 (PMC8323124; doi:10.1016/S1473-3099(20)30978-6)
Supplement: Supplementary appendix [file mmc1.pdf]

THE LANCET
Infectious Diseases

Supplementary appendix

This appendix formed part of the original submission and has been peer reviewed. We post it as supplied by the authors.

Supplement to: Drozd M, Pujades-Rodriguez M, Lillie PJ, et al. Non-communicable disease, sociodemographic factors, and risk of death from infection: a UK Biobank observational cohort study. *Lancet Infect Dis* 2021; published online March 1. https://doi.org/10.1016/S1473-3099(20)30978-6.

Supplemental Material

Supplemental Table 1: Co-morbidity definitions

NCD group	UK Biobank self-reported illnesses included
Hypertension	Hypertension<br>Essential hypertension
Chronic cardiac disease	Angina<br>Cardiomyopathy<br>Heart attack/myocardial infarction<br>heart failure/pulmonary oedema<br>Hypertrophic cardiomyopathy<br>Coronary angioplasty (ptca) +/- stent<br>Coronary artery bypass grafts<br>Triple heart bypass
Chronic respiratory disease	Asthma<br>Bronchiectasis<br>Chronic obstructive airways disease/COPD<br>Emphysema<br>Emphysema/chronic bronchitis<br>Fibrosing alveolitis/unspecified alveolitis<br>Interstitial lung disease<br>Other chronic respiratory problems<br>Pulmonary fibrosis
Diabetes	Diabetes<br>Diabetic eye disease<br>Diabetic nephropathy<br>Diabetic neuropathy/ulcers<br>Type 1 diabetes<br>Type 2 diabetes
Cancer	Any cancer diagnosis during lifetime
Chronic liver disease	Liver failure/cirrhosis<br>Non-infective hepatitis<br>Oesophageal varices<br>Primary biliary cirrhosis
Chronic kidney disease	Diabetic nephropathy<br>Immunoglobulin A (IgA) nephropathy<br>Kidney nephropathy<br>Polycystic kidney<br>Renal failure not requiring dialysis<br>Renal failure requiring dialysis<br>Renal/kidney failure
Prior stroke/TIA	Brain haemorrhage<br>Ischaemic stroke<br>Stroke<br>Subarachnoid haemorrhage<br>Transient ischaemic attack (TIA)
Other neurology disease	Cerebral palsy<br>Epilepsy<br>Motor neurone disease<br>Multiple sclerosis<br>Myasthenia gravis<br>Parkinson's disease
Psychiatric disease	Depression<br>Mania/bipolar disorder/manic depression<br>Postnatal depression<br>Schizophrenia
Chronic inflammatory and autoimmune rheumatic disease	Ankylosing spondylitis<br>Dermatomyositis

(Rheumatological disease)	Dermatopolymyositis<br>Giant cell/temporal arteritis<br>Myositis/myopathy<br>Polymyalgia rheumatica<br>Polymyositis<br>Psoriatic arthropathy<br>Rheumatoid arthritis<br>Sarcoidosis<br>Scleroderma/systemic sclerosis<br>Sjogren's syndrome/sicca syndrome<br>Systemic lupus erythematosus<br>Vasculitis

All comorbidities are defined using self-reported illness at verbal nurse led interview (UK Biobank data fields 20002 and 20004). COPD, chronic obstructive pulmonary disease.

Supplemental Table 2: ICD-10 infection-death codes

A00.0, A00.1, A00.9, A01.0-A01.4, A02.0-A02.2, A02.8, A02.9, A03.0-A03.3, A03.8, A03.9, A04.0-A04.9, A06.2, A06.4-A06.6, A07.0, A07.8, A07.9, A08.0-A08.4, A09.9, A15.0-A15.9, A16.0-A16.9, A17.0, A17.1, A17.8, A17.9, A18.0-A18.8, A19.0-A19.2, A19.8, A19.9, A20.0-A20.3, A20.7-A20.9, A21.0-A21.3, A21.7-A21.9, A22.0-A22.2, A22.7-A22.9, A23.0-A23.3, A23.8, A23.9, A24.0-A24.4, A25.0, A25.9, A26.7-A26.9, A27.0, A27.8, A27.9, A28.8, A28.9, A30.0-A30.5, A30.8, A30.9, A31.0, A31.1, A31.8, A31.9, A32.0, A32.1, A32.7-A32.9, A33.X, A34.X, A35.X, A36.0-A36.3, A36.8, A36.9, A37.0, A37.1, A37.8, A37.9, A39.0, A39.2-A39.5, A39.8, A39.9, A40.0-A40.3, A40.8, A40.9, A41.0, A41.0-A41.5, A41.8, A41.9, A42.0-A42.2, A42.7-A42.9, A46.X, A48.0, A48.8, A49.0-A49.3, A49.2, A49.8, A49.9, A54.0, A54.2-A54.4, A54.5, A54.6, A54.8, A54.9, A56.4, A57.X, A66.0-A66.4, A66.6-A66.9, A67.0-A67.3, A67.9, A68.0, A68.1, A68.9, A69.1, A70.X, A71.0, A71.1, A71.9, A74.0, A74.8, A74.9, A75.0-A75.3, A75.9, A77.0, A79.0, A79.8, A79.9, A80.9, A81.0, A81.8, A81.9, A82.0, A82.1, A82.9, A83.0, A83.6, A83.8, A83.9, A84.0, A84.8, A84.9, A85.0-A85.2, A85.8, A86.X, A87.0, A87.1, A87.2, A87.8, A87.9, A88.0, A88.8, A89.X, A90.X, A91.X, A92.0, A92.3, A92.8, A92.9, A93.0, A93.8, A94.X, A95.0, A95.1, A95.9, A96.0, A96.8, A96.9, A98.0, A98.3, A98.4, A98.8, A99.X, B00.0, B00.1, B00.2, B00.3-B00.5, B00.7-B00.9, B01.0, B01.1, B01.2, B01.8, B01.9, B02.0, B02.1, B02.2, B02.3, B02.7-B02.9, B03.X, B05.0, B05.1, B05.2, B05.3, B05.4, B05.8, B05.9, B06.0, B06.8, B06.9, B07.X, B08.0, B08.4, B08.5, B08.8, B09.X, B16.0-B16.2, B16.9, B17.0, B17.1, B17.8, B17.9, B18.0-B18.2, B18.8, B18.9, B19.0, B19.9, B25.0, B25.1, B25.2, B25.8, B25.9, B26.0, B26.1, B26.2, B26.3, B26.8, B26.9, B27.0, B27.1, B27.8, B27.9, B30.0-B30.2, B30.3, B30.8, B30.9, B33.0, B33.2-B33.4, B33.8, B34.0-B34.4 (exclude corona), B34.8, B34.9, B35.9, B36.8, B37.0-B37.9, B38.0-B38.4, B38.7-B38.9, B41.0, B41.7-B41.9, B43.1, B43.2, B44.0-B44.2, B44.7-B44.9, B45.0-B45.3, B45.7-B45.9, B46.8, B47.1, B48.7, B48.8, B50.0, B50.8, B50.9, B51.0, B51.8, B51.9, B52.0, B52.8, B52.9, B53.0, B53.1, B53.8, B54.X, B55.0-B55.2, B55.9, B56.0, B56.1, B56.9, B58.0-B58.3, B58.8, B58.9, B60.0, B60.8, B64.X, B65.0-B65.2, B65.8, B65.9, B67.8, B67.9, B71.0, B71.8, B71.9, B76.1, B81.0, B81.4, B81.8, B82.0, B82.9, B83.0, B83.8, B83.9, B85.0-B85.2, B85.4, B87.0-B87.4, B87.8, B87.9, B88.0, B89.X, B91.X, B92.X, B94.0, B94.0, B94.1, B94.2, B94.8, B94.9, B95.0-B95.8, B96.0-B96.2, B96.3, B96.4, B96.5, B96.7, B96.8, B97.0-B97.8, D47.5, D73.3, E32.1, G00.0-G00.3, G00.8, G00.9, G01.X, G02.0, G02.1, G02.8, G03.0-G03.2, G03.8, G03.9, G04.2, G05.0, G05.1, G05.2, G06.0-G06.2, G07X, G14.X, G53.0, G53.1, G63.0, G73.1, G73.4, G93.3, G94.0, H03.0, H06.1, H10.0, H10.2, H10.3, H10.4, H10.5, H10.8, H10.9, H13.0, H13.1, H13.2, H16.1, H16.2, H19.1, H19.2, H19.3, H22.0, H32.0, H60.0, H60.1, H60.2, H60.3, H60.5, H60.8, H60.9, H62.0, H62.1, H62.3, H62.4, H65.0, H65.1, H65.2-H65.4, H65.9, H66.0-H66.4, H66.9, H67.0, H67.1, H67.8, H70.0, H70.1, H70.8, H70.9, H73.0, H73.1, H75.0, H94.0, I32.0, I32.1, I41.0, I41.1, I41.2, I42.3, I43.0, I52.0, I52.1, I68.1, I98.1, J00.X, J01.0-J01.4, J01.8, J01.9, J02.0, J02.8, J02.9, J03.0, J03.0, J03.8, J03.9, J06.0, J06.8, J06.9, J09.X, J10.0, J10.0, J10.1, J10.8, J11.0, J11.1, J11.8, J12.0, J12.1, J12.2, J12.3, J12.8, J12.9, J13X, J14X, J15.0- J15.9, J16.0, J16.8, J17.0, J17.1, J17.2, J17.3, J17.8, J18.0-J18.2, J18.8, J18.9, J20.0, J20.1, J20.2, J20.3, J20.4, J20.5-J20.7, J21.0, J21.1, J22.X, J31.0-J31.2, J32.0-J32.4, J32.8, J32.9, J34.0, J35.0, J36X, J39.0, J39.1, J44.0, J65X, J85.0, J85.1, J85.2, J85.3, K04.6, K04.7, K11.3, K12.2, K23.0, K35.2, K35.3, K35.8, K36.X, K37.X, K51.5, K52. 3, K57.0, K57.1, K57.2, K57.3, K57.4, K57.5, K57.8, K57.9, K61.0-K61.4, K63.0, K65.0, K65.8, K65.9, K67.0-K67.3, K75.0, K77.0, K80.0-K80.5, K81.0, K81.1, K81.9, K83.0, K93.0, L00.X, L02.0-L02.4, L02.8, L02.9, L03.0-L03.3, L03.8, L03.9, L05.0, L05.9, L92.2, L98.3, M00.0-M00.2, M00.8, M01, M01.0, M01.1, M01.3, M01.4, M01.5, M01.6, M02.3, M03.0, M03.1, M35.4, M46.2, M49.0-M49.2, M49.3, M63.0, M63.1, M65.0, M68.0, M71.0, M73.0, M86.0-M86.2, M86.3-M86.6, M86.8, M86.9, M89.6, N08.0, N15.1, N16.0, N22.0, N29.1, N33.0, N34.0, N35.1, N39.0, N41.2, N45.0, N45.9, N73.0, N73.1, N73.2, N73.3, N73.4, N73.5, N74.0, N74.1, N75.1, N76.4, N77.0, N77.1, O26.4, O35.3, O75.3, O98.0, O98.4, O98.5, O98.6, O98.8, O98.9, R57.2

ICD-10 codes adapted from Wu J *et al* (Incidence of infections associated with oral glucocorticoid dose in people diagnosed with polymyalgia rheumatica or giant cell arteritis: a cohort study in England. CMAJ; 2019; 191(25): E680-E688) with additional inclusion of codes I33, I38, I39 (for infective endocarditis) and removal of code symptoms (R05, R07.0). A full description of each code is provided on pages 17-43 of this document.

Supplemental Table 3: Classification of infection deaths

Infection classification	Number (%)
Blood stream	81 (5.9%)
Bone, joint and connective tissue	5 (0.4%)
Digestive tract, including liver	196 (14.2%)
Genitourinary	82 (5.9%)
Heart and circulation	61 (4.4%)
Lower respiratory tract	840 (60.6%)
Neurological and eye	59 (4.3%)
Other infections	38 (2.7%)
Skin and soft tissue	20 (1.4%)
Upper respiratory tract, including ENT	3 (0.2%)

Data illustrated Figure 1b. ENT denotes ear, nose and throat.

Supplemental Table 4: Incidence rate ratios and 95% confidence intervals from unadjusted, age/sex, age/sex/demographic and fully adjusted Poisson models for infection and non-infection death

	Infection Death IRR (95% CI)				Non-infection Death IRR (95% CI)			
**Model**	**Crude**	**Age/Sex**	**Age/Sex/ Demographic**	**Full**	**Crude**	**Age/Sex**	**Age/Sex/ Demographic**	**Full**
**Age (reference: 45)**								
**50**	1.68 (1.33-2.12)	1.69 (1.34-2.14)	1.75 (1.39-2.21)	1.64 (1.30-2.08)	1.59 (1.52-1.65)	1.60 (1.54-1.67)	1.63 (1.57-1.70)	1.55 (1.49-1.62)
**55**	3.26 (2.51-4.23)	3.30 (2.54-4.28)	3.55 (2.74-4.61)	3.13 (2.41-4.07)	2.42 (2.30-2.55)	2.44 (2.32-2.58)	2.53 (2.40-2.67)	2.29 (2.18-2.42)
**60**	5.10 (3.95-6.60)	5.12 (3.96-6.62)	5.68 (4.39-7.36)	4.77 (3.68-6.19)	3.68 (3.50-3.87)	3.69 (3.51-3.88)	3.86 (3.67-4.06)	3.32 (3.16-3.49)
**65**	8.43 (6.61-10.76)	8.32 (6.53-10.62)	9.53 (7.46-12.18)	7.59 (5.92-9.73)	6.15 (5.87-6.45)	6.08 (5.80-6.38)	6.47 (6.16-6.79)	5.21 (4.97-5.48)
**Sex (reference: female)**								
**Male**	1.88 (1.69-2.10)	1.80 (1.61-2.00)	1.62 (1.46-1.81)	1.60 (1.43-1.79)	1.79 (1.75-1.84)	1.73 (1.69-1.77)	1.61 (1.57-1.65)	1.61 (1.57-1.65)
**Ethnicity (reference: white)**								
**BAME**	0.56 (0.41-0.76)	0.81 (0.59-1.11)	0.68 (0.49-0.93)	0.64 (0.46-0.87)	0.64 (0.60-0.69)	0.89 (0.83-0.95)	0.82 (0.77-0.88)	0.80 (0.75-0.86)
**SED Quintile (reference: 1)**								
**2**	1.22 (1.00-1.49)	1.22 (0.99-1.49)	1.19 (0.97-1.46)	1.17 (0.95-1.43)	1.03 (0.98-1.07)	1.02 (0.98-1.07)	1.01 (0.97-1.05)	1.00 (0.96-1.04)
**3**	1.42 (1.17-1.73)	1.46 (1.20-1.78)	1.40 (1.15-1.70)	1.32 (1.09-1.61)	1.09 (1.05-1.14)	1.12 (1.08-1.17)	1.09 (1.05-1.13)	1.06 (1.02-1.10)
**4**	1.76 (1.46-2.13)	1.94 (1.61-2.35)	1.78 (1.47-2.15)	1.60 (1.33-1.94)	1.17 (1.13-1.22)	1.28 (1.23-1.33)	1.21 (1.16-1.25)	1.14 (1.09-1.19)
**5**	2.85 (2.39-3.40)	3.31 (2.78-3.95)	2.74 (2.29-3.27)	2.13 (1.78-2.56)	1.56 (1.51-1.62)	1.79 (1.72-1.86)	1.57 (1.52-1.64)	1.38 (1.33-1.43)
**Smoking (reference: never)**								
**Former**	2.12 (1.87-2.40)	1.63 (1.44-1.85)	1.54 (1.36-1.75)	1.38 (1.22-1.57)	1.77 (1.72-1.82)	1.40 (1.36-1.44)	1.36 (1.32-1.40)	1.27 (1.24-1.31)
**Current**	4.41 (3.83-5.06)	4.76 (4.14-5.48)	3.95 (3.42-4.56)	3.69 (3.19-4.26)	2.71 (2.62-2.81)	2.85 (2.76-2.95)	2.61 (2.52-2.70)	2.52 (2.44-2.61)
**Obesity (reference: non-obese)**								
**Class 1**	1.30 (1.13-1.48)	1.20 (1.05-1.38)	1.15 (1.00-1.31)	0.95 (0.83-1.09)	1.29 (1.25-1.33)	1.20 (1.17-1.24)	1.17 (1.14-1.21)	1.06 (1.03-1.09)

**Class 2**	1.56 (1.26-1.92)	1.63 (1.32-2.02)	1.49 (1.21-1.84)	1.04 (0.84-1.30)	1.51 (1.44-1.59)	1.57 (1.49-1.65)	1.49 (1.42-1.57)	1.23 (1.17-1.29)
**Class 3**	3.37 (2.68-4.23)	4.31 (3.43-5.43)	3.79 (3.01-4.77)	2.21 (1.74-2.82)	1.80 (1.68-1.93)	2.21 (2.06-2.37)	2.06 (1.92-2.21)	1.55 (1.44-1.66)
**Hypertension**	2.62 (2.36-2.91)	1.79 (1.61-2.00)	1.73 (1.56-1.93)	1.36 (1.22-1.53)	1.93 (1.88-1.97)	1.37 (1.34-1.41)	1.35 (1.32-1.38)	1.15 (1.12-1.18)
**Chronic cardiac disease**	4.62 (4.03-5.29)	2.61 (2.26-3.00)	2.27 (1.97-2.61)	1.66 (1.44-1.92)	3.26 (3.15-3.38)	1.95 (1.88-2.02)	1.80 (1.73-1.87)	1.54 (1.48-1.60)
**Chronic respiratory disease**	2.61 (2.32-2.94)	2.68 (2.38-3.02)	2.49 (2.21-2.80)	2.21 (1.96-2.50)	1.38 (1.33-1.42)	1.42 (1.37-1.46)	1.36 (1.32-1.41)	1.28 (1.24-1.32)
**Diabetes**	3.89 (3.38-4.49)	2.69 (2.33-3.11)	2.47 (2.13-2.85)	1.78 (1.52-2.07)	2.85 (2.75-2.95)	2.05 (1.98-2.13)	1.95 (1.88-2.03)	1.61 (1.54-1.67)
**Cancer**	1.70 (1.45-1.99)	1.33 (1.14-1.56)	1.31 (1.12-1.54)	1.30 (1.11-1.52)	2.72 (2.64-2.80)	2.25 (2.18-2.32)	2.23 (2.16-2.30)	2.22 (2.16-2.30)
**Chronic liver disease**	4.70 (2.66-8.29)	4.63 (2.62-8.17)	3.74 (2.12-6.60)	3.21 (1.81-5.67)	3.02 (2.56-3.55)	2.96 (2.52-3.49)	2.62 (2.23-3.09)	2.37 (2.01-2.78)
**Chronic kidney disease**	9.02 (6.25-13.04)	7.80 (5.40-11.26)	7.46 (5.16-10.78)	5.04 (3.48-7.31)	3.90 (3.43-4.42)	3.43 (3.02-3.89)	3.35 (2.95-3.81)	2.50 (2.20-2.84)
**Prior stroke/TIA**	4.79 (3.92-5.85)	3.07 (2.51-3.76)	2.61 (2.13-3.19)	1.87 (1.52-2.30)	3.00 (2.83-3.17)	2.03 (1.92-2.15)	1.85 (1.75-1.96)	1.51 (1.43-1.60)
**Other neurology disease**	3.27 (2.51-4.27)	3.30 (2.53-4.30)	3.09 (2.37-4.03)	2.84 (2.17-3.71)	2.27 (2.12-2.44)	2.29 (2.13-2.46)	2.21 (2.05-2.37)	2.13 (1.98-2.29)
**Psychiatric disorder**	1.78 (1.50-2.12)	2.24 (1.88-2.67)	1.83 (1.54-2.19)	1.56 (1.30-1.86)	1.24 (1.19-1.30)	1.51 (1.44-1.58)	1.34 (1.28-1.40)	1.23 (1.18-1.29)
**Rheumatological disease**	3.31 (2.69-4.07)	3.02 (2.46-3.72)	2.83 (2.30-3.48)	2.45 (1.99-3.02)	1.68 (1.58-1.79)	1.56 (1.46-1.66)	1.50 (1.40-1.59)	1.41 (1.32-1.51)

Demographic refers to ethnicity, SED and smoking status. Fully adjusted refers to age, sex, demographics and all listed non-communicable diseases. BAME – black, Asian and minority ethnicity; CI – confidence interval; IRR – incidence rate ratio; SED – socioeconomic deprivation; TIA – transient ischaemic attack.

Supplemental Table 5: Incidence rate ratios and 95% confidence intervals from fully adjusted Poisson models for infection and non-infection death with minority ethnic groups considered individually.

	Infection death IRR (95% CI)	Non-infection death IRR (95% CI)
**Age (reference: 45)**		
**50**	1.64 (1.30-2.08)	1.55 (1.49-1.62)
**55**	3.13 (2.41-4.06)	2.29 (2.18-2.42)
**60**	4.76 (3.67-6.18)	3.32 (3.16-3.49)
**65**	7.58 (5.91-9.72)	5.22 (4.97-5.48)
**Sex (reference: female)**		
**Male**	1.60 (1.43-1.79)	1.61 (1.57-1.65)
**Ethnicity (reference: white)**		
**Mixed**	0.58 (0.22-1.55)	0.88 (0.72-1.06)
**Asian**	0.74 (0.47-1.17)	0.78 (0.70-0.86)
**Black**	0.63 (0.36-1.12)	0.76 (0.67-0.87)
**Chinese**	0.47 (0.07-3.32)	0.79 (0.58-1.10)
**Other**	0.46 (0.19-1.12)	0.87 (0.74-1.01)
**SED quintile (reference: 1)**		
**2**	1.17 (0.95-1.43)	1.00 (0.96-1.04)
**3**	1.32 (1.09-1.61)	1.06 (1.02-1.10)
**4**	1.60 (1.33-1.94)	1.14 (1.10-1.19)
**5**	2.13 (1.78-2.56)	1.38 (1.33-1.44)
**Smoking (reference: never)**		
**Former**	1.38 (1.22-1.57)	1.27 (1.24-1.31)
**Current**	3.70 (3.20-4.27)	2.52 (2.43-2.61)
**Obesity (reference: non-obese)**		
**Class 1**	0.95 (0.83-1.09)	1.06 (1.03-1.09)
**Class 2**	1.05 (0.84-1.30)	1.23 (1.17-1.29)
**Class 3**	2.22 (1.74-2.82)	1.55 (1.44-1.66)
**Hypertension**	1.36 (1.22-1.53)	1.15 (1.12-1.18)
**Chronic cardiac disease**	1.66 (1.44-1.92)	1.54 (1.48-1.60)
**Chronic respiratory disease**	2.21 (1.96-2.49)	1.28 (1.24-1.32)
**Diabetes**	1.77 (1.52-2.07)	1.61 (1.55-1.67)
**Cancer**	1.30 (1.11-1.52)	2.22 (2.16-2.29)
**Chronic liver disease**	3.21 (1.82-5.67)	2.37 (2.01-2.78)
**Chronic kidney disease**	5.04 (3.48-7.31)	2.50 (2.20-2.84)
**Prior stroke/TIA**	1.87 (1.52-2.30)	1.51 (1.43-1.60)
**Other neurology disease**	2.84 (2.18-3.71)	2.13 (1.98-2.29)
**Psychiatric disorder**	1.56 (1.30-1.86)	1.23 (1.18-1.29)

**Rheumatological disease**	2.45 (1.99-3.01)	1.41 (1.32-1.51)

Fully adjusted refers to age, sex, demographics and all listed non-communicable diseases. CI – confidence interval; IRR – incidence rate ratio; SED – socioeconomic deprivation; TIA – transient ischaemic attack.

Supplemental Table 6: Incidence rate ratios and 95% confidence intervals from fully adjusted Poisson models for lower respiratory tract infection death and other infection death.

	LRTI death<br>IRR (95% CI)	Other infection death<br>IRR (95% CI)
**Age (reference: 45)**		
**50**	1.64 (1.20-2.24)	1.64 (1.15-2.33)
**55**	3.60 (2.55-5.10)	2.55 (1.71-3.79)
**60**	5.66 (4.00-8.02)	3.73 (2.52-5.52)
**65**	8.91 (6.39-12.43)	5.99 (4.12-8.73)
**Sex (reference: female)**		
**Male**	1.82 (1.57-2.10)	1.33 (1.11-1.58)
**Ethnicity (reference: white)**		
**BAME**	0.58 (0.38-0.88)	0.73 (0.45-1.17)
**SED quintile (reference: 1)**		
**2**	1.18 (0.89-1.55)	1.16 (0.86-1.57)
**3**	1.38 (1.06-1.80)	1.26 (0.94-1.69)
**4**	1.82 (1.42-2.34)	1.34 (1.00-1.80)
**5**	2.51 (1.97-3.18)	1.67 (1.26-2.21)
**Smoking (reference: never)**		
**Former**	1.57 (1.32-1.86)	1.18 (0.97-1.42)
**Current**	4.76 (3.95-5.73)	2.43 (1.91-3.09)
**Obesity (reference: non-obese)**		
**Class 1**	0.76 (0.63-0.91)	1.30 (1.06-1.60)
**Class 2**	0.91 (0.69-1.21)	1.30 (0.93-1.81)
**Class 3**	1.96 (1.44-2.68)	2.69 (1.83-3.94)
**Hypertension**	1.34 (1.16-1.55)	1.40 (1.17-1.68)
**Chronic cardiac disease**	1.55 (1.28-1.86)	1.86 (1.47-2.36)
**Chronic respiratory disease**	2.89 (2.50-3.35)	1.35 (1.08-1.67)
**Diabetes**	1.91 (1.57-2.32)	1.59 (1.24-2.05)
**Cancer**	1.13 (0.91-1.40)	1.57 (1.24-2.00)
**Chronic liver disease**	1.72 (0.64-4.60)	5.75 (2.85-11.59)
**Chronic kidney disease**	4.43 (2.65-7.40)	6.03 (3.52-10.31)
**Prior stroke/TIA**	1.87 (1.45-2.41)	1.86 (1.32-2.62)
**Other neurology disease**	3.49 (2.56-4.76)	1.81 (1.06-3.08)
**Psychiatric disorder**	1.76 (1.42-2.18)	1.23 (0.90-1.68)
**Rheumatological disease**	2.22 (1.69-2.93)	2.81 (2.05-3.86)

Fully adjusted refers to age, sex, demographics and all listed non-communicable diseases. BAME – black, Asian and minority ethnicity; CI – confidence interval; IRR – incidence rate ratio; LRTI – lower respiratory tract infection; SED – socioeconomic deprivation; TIA – transient ischaemic attack.

Supplemental Table 7: Incidence rate ratios and 95% confidence intervals from fully adjusted Poisson models including only events within first 5- or 9-years follow-up.

	Infection death IRR (95% CI)		Non-infection death IRR (95% CI)	
**Timepoint**	**5 years**	**9 years**	**5 years**	**9 years**
**Age (reference: 45)**				
**50**	1.80 (1.11-2.94)	1.50 (1.15-1.96)	1.54 (1.43-1.65)	1.54 (1.47-1.61)
**55**	3.57 (2.07-6.15)	2.81 (2.08-3.79)	2.11 (1.93-2.31)	2.23 (2.10-2.37)
**60**	4.21 (2.45-7.24)	4.25 (3.14-5.76)	2.72 (2.49-2.97)	3.14 (2.96-3.33)
**65**	5.12 (3.02-8.69)	6.48 (4.86-8.64)	4.01 (3.68-4.36)	4.85 (4.58-5.13)
**Sex (reference: female)**				
**Male**	1.56 (1.22-1.99)	1.61 (1.40-1.85)	1.74 (1.66-1.82)	1.64 (1.59-1.69)
**Ethnicity (reference: white)**				
**BAME**	0.59 (0.29-1.20)	0.64 (0.44-0.93)	0.70 (0.62-0.80)	0.82 (0.76-0.89)
**SED quintile (reference: 1)**				
**2**	1.20 (0.78-1.86)	1.22 (0.94-1.57)	1.01 (0.94-1.09)	1.02 (0.98-1.07)
**3**	1.12 (0.72-1.74)	1.38 (1.07-1.76)	1.10 (1.03-1.19)	1.09 (1.04-1.14)
**4**	1.72 (1.15-2.59)	1.81 (1.43-2.30)	1.22 (1.14-1.31)	1.17 (1.12-1.23)
**5**	2.07 (1.40-3.08)	2.25 (1.79-2.83)	1.48 (1.38-1.58)	1.40 (1.34-1.47)
**Smoking (reference: never)**				
**Former**	1.48 (1.13-1.94)	1.31 (1.13-1.54)	1.28 (1.21-1.34)	1.28 (1.24-1.32)
**Current**	2.81 (2.02-3.91)	3.39 (2.83-4.04)	2.48 (2.33-2.64)	2.54 (2.44-2.65)
**Obesity (reference: non-obese)**				
**Class 1**	0.84 (0.62-1.15)	0.85 (0.71-1.01)	1.03 (0.98-1.09)	1.04 (1.00-1.08)
**Class 2**	0.89 (0.54-1.47)	0.96 (0.73-1.26)	1.18 (1.08-1.30)	1.21 (1.14-1.28)
**Class 3**	1.86 (1.07-3.23)	1.92 (1.42-2.60)	1.46 (1.28-1.66)	1.52 (1.39-1.65)
**Hypertension**	1.45 (1.12-1.86)	1.39 (1.21-1.60)	1.08 (1.02-1.13)	1.15 (1.11-1.19)
**Chronic cardiac disease**	1.62 (1.17-2.26)	1.66 (1.39-1.99)	1.69 (1.58-1.80)	1.55 (1.49-1.62)
**Chronic respiratory disease**	2.30 (1.77-2.99)	2.38 (2.06-2.75)	1.28 (1.21-1.36)	1.29 (1.24-1.34)
**Diabetes**	1.32 (0.91-1.92)	1.82 (1.50-2.19)	1.65 (1.54-1.77)	1.61 (1.54-1.69)
**Cancer**	1.56 (1.12-2.16)	1.32 (1.09-1.60)	3.52 (3.35-3.70)	2.49 (2.41-2.58)
**Chronic liver disease**	5.04 (1.87-13.57)	3.56 (1.84-6.88)	2.73 (2.10-3.55)	2.51 (2.09-3.02)
**Chronic kidney disease**	6.92 (3.53-13.58)	6.54 (4.41-9.71)	2.65 (2.14-3.27)	2.70 (2.34-3.11)
**Prior stroke/TIA**	2.52 (1.67-3.80)	2.16 (1.70-2.74)	1.54 (1.39-1.71)	1.49 (1.40-1.60)
**Other neurology disease**	2.93 (1.67-5.13)	2.71 (1.95-3.78)	1.98 (1.74-2.26)	2.06 (1.89-2.24)
**Psychiatric disorder**	1.61 (1.10-2.36)	1.57 (1.26-1.94)	1.24 (1.14-1.34)	1.24 (1.17-1.31)
**Rheumatological disease**	1.77 (1.05-2.99)	2.28 (1.75-2.97)	1.40 (1.25-1.57)	1.39 (1.29-1.50)

Fully adjusted refers to age, sex, demographics and all listed non-communicable diseases. BAME – black, Asian and minority ethnicity; CI – confidence interval; IRR – incidence rate ratio; SED – socioeconomic deprivation; TIA – transient ischaemic attack.

Supplemental Table 8: Incidence rate ratios and 95% confidence intervals from age-sex adjusted Poisson models using study cohort versus main UK biobank cohort.

	Infection Death IRR (95% CI)		Non-infection Death IRR (95% CI)	
**Cohort**	**Study cohort**	**Complete UK Biobank cohort**	**Study cohort**	**Complete UK Biobank cohort**
**Cohort size**	493,295	502,505	493,295	502,505
**Events**	1,385	1441	26,344	27,105
**Age (reference: 45)**				
**50**	1.69 (1.34-2.14)	1.71 (1.36-2.15)	1.60 (1.54-1.67)	1.60 (1.53-1.66)
**55**	3.30 (2.54-4.28)	3.28 (2.54-4.22)	2.44 (2.32-2.58)	2.44 (2.32-2.57)
**60**	5.12 (3.96-6.62)	4.97 (3.87-6.39)	3.69 (3.51-3.88)	3.68 (3.51-3.87)
**65**	8.32 (6.53-10.62)	8.09 (6.39-10.26)	6.08 (5.80-6.38)	6.05 (5.77-6.33)
**Sex (reference: female)**				
**Male**	1.80 (1.61-2.00)	1.78 (1.60-1.98)	1.73 (1.69-1.77)	1.72 (1.68-1.77)
**Ethnicity (reference: white)**				
**BAME**	0.81 (0.59-1.11)	0.86 (0.65-1.13)	0.89 (0.83-0.95)	0.94 (0.89-1.00)
**SED quintile (reference: 1)**				
**2**	1.22 (0.99-1.49)	1.26 (1.03-1.54)	1.02 (0.98-1.07)	1.03 (0.99-1.08)
**3**	1.46 (1.20-1.78)	1.54 (1.27-1.87)	1.12 (1.08-1.17)	1.12 (1.08-1.17)
**4**	1.94 (1.61-2.35)	1.98 (1.64-2.39)	1.28 (1.23-1.33)	1.29 (1.24-1.34)
**5**	3.31 (2.78-3.95)	3.49 (2.94-4.16)	1.79 (1.72-1.86)	1.81 (1.74-1.88)
**Smoking (reference: never)**				
**Former**	1.63 (1.44-1.85)	1.64 (1.45-1.86)	1.40 (1.36-1.44)	1.40 (1.36-1.44)
**Current**	4.76 (4.14-5.48)	4.75 (4.14-5.46)	2.85 (2.76-2.95)	2.86 (2.76-2.95)
**Obesity (reference: non-obese)**				
**Class 1**	1.20 (1.05-1.38)	1.2 (1.05-1.37)	1.20 (1.17-1.24)	1.20 (1.17-1.24)
**Class 2**	1.63 (1.32-2.02)	1.65 (1.34-2.03)	1.57 (1.49-1.65)	1.58 (1.50-1.65)
**Class 3**	4.31 (3.43-5.43)	4.34 (3.46-5.44)	2.21 (2.06-2.37)	2.22 (2.07-2.38)
**Hypertension**	1.79 (1.61-2.00)	1.81 (1.63-2.01)	1.37 (1.34-1.41)	1.38 (1.35-1.42)
**Chronic cardiac disease**	2.61 (2.26-3.00)	2.66 (2.32-3.06)	1.95 (1.88-2.02)	1.96 (1.89-2.03)
**Chronic respiratory disease**	2.68 (2.38-3.02)	2.72 (2.43-3.06)	1.42 (1.37-1.46)	1.42 (1.38-1.47)
**Diabetes**	2.69 (2.33-3.11)	2.73 (2.37-3.14)	2.05 (1.98-2.13)	2.07 (1.99-2.14)
**Cancer**	1.33 (1.14-1.56)	1.34 (1.14-1.56)	2.25 (2.18-2.32)	2.24 (2.17-2.31)
**Chronic liver disease**	4.63 (2.62-8.17)	4.47 (2.53-7.89)	2.96 (2.52-3.49)	2.95 (2.51-3.47)
**Chronic kidney disease**	7.80 (5.40-11.26)	7.74 (5.39-11.11)	3.43 (3.02-3.89)	3.52 (3.11-3.98)
**Prior stroke/TIA**	3.07 (2.51-3.76)	3.24 (2.67-3.92)	2.03 (1.92-2.15)	2.06 (1.95-2.17)
**Other neurology disease**	3.30 (2.53-4.30)	3.46 (2.69-4.44)	2.29 (2.13-2.46)	2.44 (2.28-2.61)
**Psychiatric disorder**	2.24 (1.88-2.67)	2.34 (1.98-2.77)	1.51 (1.44-1.58)	1.51 (1.45-1.58)

**Rheumatological disease**	3.02 (2.46-3.72)	3.00 (2.45-3.68)	1.56 (1.46-1.66)	1.58 (1.48-1.68)

BAME – black, Asian and minority ethnicity; CI – confidence interval; IRR – incidence rate ratio; SED – socioeconomic deprivation; TIA – transient ischaemic attack.

Supplemental Table 9: Incidence rate ratios and 95% confidence intervals from unadjusted, age/sex and age/sex/demographic adjusted Poisson models for infection and non-infection death considering non-communicable disease defined as number of co-morbidities.

	Infection Death IRR (95% CI)			Non-infection Death IRR (95% CI)		
**Model**	**Crude**	**Age/Sex**	**Age/Sex/ Demographic**	**Crude**	**Age/Sex**	**Age/Sex/ Demographic**
**Comorbidities (reference: none)**						
**1**	2.09 (1.77-2.46)	1.76 (1.49-2.08)	1.70 (1.44-2.00)	1.72 (1.67-1.78)	1.48 (1.43-1.53)	1.45 (1.41-1.50)
**2**	4.41 (3.74-5.19)	3.26 (2.76-3.85)	3.01 (2.55-3.56)	2.67 (2.58-2.77)	2.04 (1.97-2.11)	1.96 (1.89-2.03)
**3**	7.84 (6.55-9.39)	5.33 (4.44-6.39)	4.71 (3.92-5.65)	3.93 (3.77-4.10)	2.78 (2.67-2.90)	2.61 (2.50-2.72)
**4**	13.43 (10.77-16.73)	8.53 (6.83-10.65)	7.05 (5.63-8.82)	6.11 (5.78-6.46)	4.08 (3.86-4.32)	3.68 (3.47-3.89)
**5+**	19.86 (14.58-27.05)	12.59 (9.23-17.17)	9.53 (6.97-13.03)	9.19 (8.46-9.98)	6.12 (5.63-6.64)	5.26 (4.84-5.72)

Demographic refers to ethnicity, SED and smoking status. IRR – incidence rate ratio.

Supplemental Table 10: Prevalence of NCDs in UKB versus a representative sample of the UK population

	Men				Women			
	Age 45–54 Years		Age 55–64 Years		Age 45–54 Years		Age 55–64 Years	
	UK Biobank	HSE	UK Biobank	HSE	UK Biobank	HSE	UK Biobank	HSE
Chronic cardiac disease	6.3	8.1	17.7	24.9	1.9	3.2	5.6	8.3
Stroke	0.8	1.2	1.9	3	0.6	0.9	1	2.3
Hypertension	21.2	27	34.4	39	15.4	16	27.4	29
Diabetes	4.5	8.1	7.8	10.5	2.4	3.5	6.3	8
Chronic kidney disease	0.2	1.1	0.3	1.5	0.2	1.2	0.2	1.9
Chronic respiratory disease	11.8	13	10.3	16	13.1	16	12.2	17

Adapted from Fry et al (Am J Epidemiol 2017; 186(9): 1026–1034). Chronic cardiac disease data were derived by summing ischaemic heart disease, angina and myocardial infarction. Chronic respiratory disease was derived by summing Asthma and COPD. Data represent % of study population with self-reported disease. HSE – Health Survey for England.

Supplemental Figure 1: Association of age with infection death or non-infection death using Poisson regression and age modelled using restricted cubic splines.

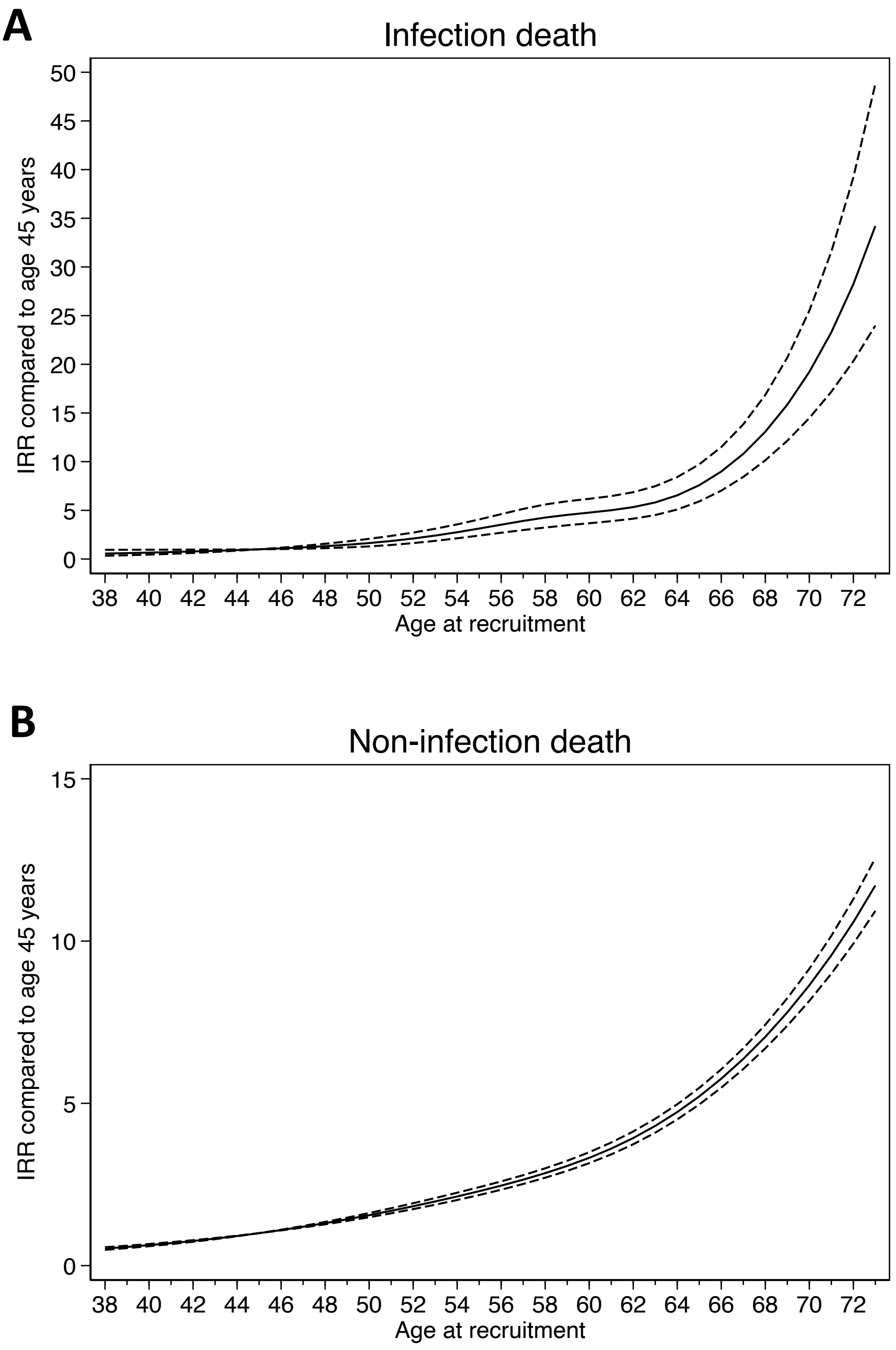

Incidence rate ratios (IRR) for infection death (panel A) or non-infection death (panel B) using Poisson regression (fully adjusted models) and age modelled using restricted cubic splines (5 knots for infection death and 4 knots for non-infection death)

Supplemental Figure 2: Timing of infection and non-infection deaths during follow-up.

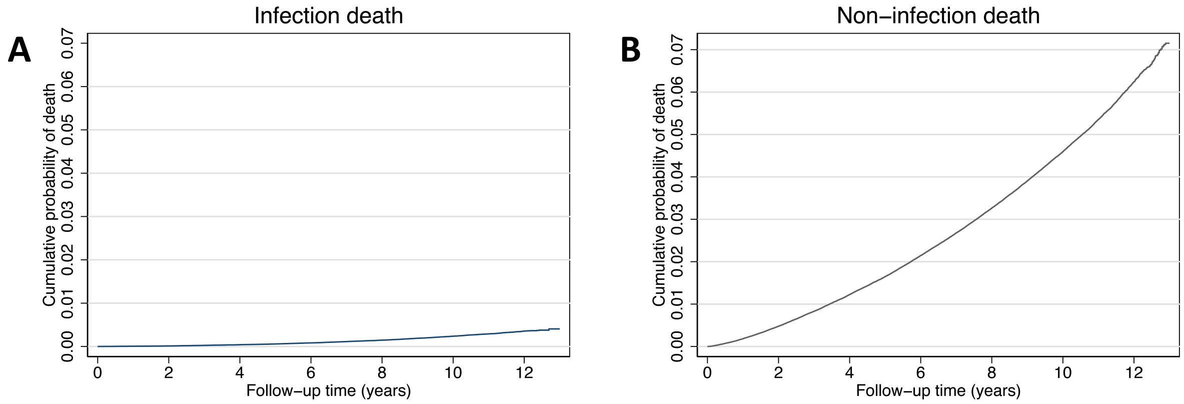

At risk	493295	490880	487055	482320	476497	387356	49015
Censored	0	2354	6039	10573	16090	104810	442903
Events	0	61	201	402	708	1129	1377

At risk	493295	490880	487055	482320	476497	387356	49015
Censored	0	61	201	402	708	83467	418126
Events	0	2354	6039	10573	16090	22472	26154

Kaplan-Meier mortality curves illustrating the cumulative probability of infection (A) or non-infection (B) death during follow-up.

Appendix A: Description of ICD10 codes in Supplemental Table 2

ICD10 code	Description
A00.0	Cholera due to Vibrio cholerae 01, biovar cholerae
A00.1	Cholera due to Vibrio cholerae 01, biovar el tor
A00.9	Cholera, unspecified
A01.0	Typhoid fever
A01.1	Paratyphoid fever A
A01.2	Paratyphoid fever B
A01.3	Paratyphoid fever C
A01.4	Paratyphoid fever, unspecified
A02.0	Salmonella gastro-enteritis
A02.1	Salmonella septicaemia
A02.2	Localised Salmonella infections
A02.8	Other specified Salmonella infections
A02.9	Salmonella infection, unspecified
A03.0	Shigellosis due to Shigella dysenteriae
A03.1	Shigellosis due to Shigella flexneri
A03.2	Shigellosis due to Shigella boydii
A03.3	Shigellosis due to Shigella sonnei
A03.8	Other shigellosis
A03.9	Shigellosis, unspecified
A04.0	Enteropathogenic Escherichia coli infection
A04.1	Enterotoxigenic Escherichia coli infection
A04.2	Enteroinvasive Escherichia coli infection
A04.3	Enterohaemorrhagic Escherichia coli infection
A04.4	Other intestinal Escherichia coli infections
A04.5	Campylobacter enteritis
A04.6	Enteritis due to Yersinia enterocolitica
A04.7	Enterocolitis due to Clostridium difficile
A04.8	Other specified bacterial intestinal infections
A04.9	Bacterial intestinal infection, unspecified
A06.2	Amoebic nondysenteric colitis
A06.4	Amoebic liver abscess
A06.5	Amoebic lung abscess
A06.6	Amoebic brain abscess
A07.0	Balantidiasis
A07.8	Other specified protozoal intestinal diseases
A07.9	Protozoal intestinal disease, unspecified
A08.0	Rotaviral enteritis
A08.1	Acute gastro-enteropathy due to Norwalk agent
A08.2	Adenoviral enteritis
A08.3	Other viral enteritis

A08.4	Viral intestinal infection, unspecified
A09.9	Gastroenteritis and colitis of unspecified origin
A15.0	Tuberculosis of lung, confirmed by sputum microscopy with or without culture
A15.1	Tuberculosis of lung, confirmed by culture only
A15.2	Tuberculosis of lung, confirmed histologically
A15.3	Tuberculosis of lung, confirmed by unspecified means
A15.4	Tuberculosis of intrathoracic lymph nodes, confirmed bacteriologically and histologically
A15.5	Tuberculosis of larynx, trachea and bronchus, confirmed bacteriologically and histologically
A15.6	Tuberculous pleurisy, confirmed bacteriologically and histologically
A15.7	Primary respiratory tuberculosis, confirmed bacteriologically and histologically
A15.8	Other respiratory tuberculosis, confirmed bacteriologically and histologically
A15.9	Respiratory tuberculosis unspecified, confirmed bacteriologically and histologically
A16	Respiratory tuberculosis, not confirmed bacteriologically or histologically
A16.0	Tuberculosis of lung, bacteriologically and histologically negative
A16.1	Tuberculosis of lung, bacteriological and histological examination not done
A16.2	Tuberculosis of lung, without mention of bacteriological or histological confirmation
A16.3	Tuberculosis of intrathoracic lymph nodes, without mention of bacteriological or histological confirmation
A16.4	Tuberculosis of larynx, trachea and bronchus, without mention of bacteriological or histological confirmation
A16.5	Tuberculous pleurisy, without mention of bacteriological or histological confirmation
A16.7	Primary respiratory tuberculosis without mention of bacteriological or histological confirmation
A16.8	Other respiratory tuberculosis, without mention of bacteriological or histological confirmation
A16.9	Respiratory tuberculosis unspecified, without mention of bacteriological or histological confirmation
A17	Tuberculosis of nervous system
A17.0	Tuberculous meningitis
A17.1	Meningeal tuberculoma
A17.8	Other tuberculosis of nervous system
A17.9	Tuberculosis of nervous system, unspecified
A18	Tuberculosis of other organs
A18.0	Tuberculosis of bones and joints
A18.1	Tuberculosis of genito-urinary system
A18.2	Tuberculous peripheral lymphadenopathy
A18.3	Tuberculosis of intestines, peritoneum and mesenteric glands
A18.4	Tuberculosis of skin and subcutaneous tissue
A18.5	Tuberculosis of eye
A18.6	Tuberculosis of ear
A18.7	Tuberculosis of adrenal glands
A19	Miliary tuberculosis
A19.0	Acute miliary tuberculosis of a single specified site
A19.1	Acute miliary tuberculosis of multiple sites
A19.2	Acute miliary tuberculosis, unspecified
A19.8	Other miliary tuberculosis

A19.9	Miliary tuberculosis, unspecified
A20	Plague
A20.0	Bubonic plague
A20.1	Cellulocutaneous plague
A20.2	Pneumonic plague
A20.3	Plague meningitis
A20.7	Septicaemic plague
A20.8	Other forms of plague
A20.9	Plague, unspecified
A21.0	Ulceroglandular tularaemia
A21.1	Oculoglandular tularaemia
A21.2	Pulmonary tularaemia
A21.3	Gastro-intestinal tularaemia
A21.7	Generalised tularaemia
A21.8	Other forms of tularaemia
A21.9	Tularaemia, unspecified
A22	Anthrax
A22.0	Cutaneous anthrax
A22.1	Pulmonary anthrax
A22.2	Gastro-intestinal anthrax
A22.7	Anthrax septicaemia
A22.8	Other forms of anthrax
A22.9	Anthrax, unspecified
A23.0	Brucellosis due to Brucella melitensis
A23.1	Brucellosis due to Brucella abortus
A23.2	Brucellosis due to Brucella suis
A23.3	Brucellosis due to Brucella canis
A23.8	Other brucellosis
A23.9	Brucellosis, unspecified
A24.0	Glanders
A24.1	Acute and fulminating melioidosis
A24.2	Subacute and chronic melioidosis
A24.3	Other melioidosis
A24.4	Melioidosis, unspecified
A25.0	Spirillosis
A25.1	Streptobacillosis
A25.9	Rat-bite fever, unspecified
A26.7	Erysipelothrix septicaemia
A26.8	Other forms of erysipeloid
A26.9	Erysipeloid, unspecified
A27.0	Leptospirosis icterohaemorrhagica
A27.8	Other forms of leptospirosis
A27.9	Leptospirosis, unspecified

A28.8	Other specified zoonotic bacterial diseases, not elsewhere classified
A28.9	Zoonotic bacterial disease, unspecified
A30.0	Indeterminate leprosy
A30.1	Tuberculoid leprosy
A30.2	Borderline tuberculoid leprosy
A30.3	Borderline leprosy
A30.4	Borderline lepromatous leprosy
A30.5	Lepromatous leprosy
A30.8	Other forms of leprosy
A30.9	Leprosy, unspecified
A31.0	Pulmonary mycobacterial infection
A31.1	Cutaneous mycobacterial infection
A31.8	Other mycobacterial infections
A31.9	Mycobacterial infection, unspecified
A32.0	Cutaneous listeriosis
A32.1	Listerial meningitis and meningoencephalitis
A32.7	Listerial septicaemia
A32.8	Other forms of listeriosis
A32.9	Listeriosis, unspecified
A33	Tetanus neonatorum
A34	Obstetrical tetanus
A35	Other tetanus
A36.0	Pharyngeal diphtheria
A36.1	Nasopharyngeal diphtheria
A36.2	Laryngeal diphtheria
A36.3	Cutaneous diphtheria
A36.8	Other diphtheria
A36.9	Diphtheria, unspecified
A37.0	Whooping cough due to Bordetella pertussis
A37.1	Whooping cough due to Bordetella parapertussis
A37.8	Whooping cough due to other Bordetella species
A37.9	Whooping cough, unspecified
A39	Meningococcal infection
A39.0	Meningococcal meningitis
A39.2	Acute meningococcaemia
A39.3	Chronic meningococcaemia
A39.4	Meningococcaemia, unspecified
A39.5	Meningococcal heart disease
A39.8	Other meningococcal infections
A39.9	Meningococcal infection, unspecified
A40.0	Septicaemia due to streptococcus, group A
A40.1	Septicaemia due to streptococcus, group B
A40.2	Septicaemia due to streptococcus, group D

A40.3	Septicaemia due to Streptococcus pneumoniae
A40.8	Other streptococcal septicaemia
A40.9	Streptococcal septicaemia, unspecified
A41.0	Septicaemia due to Staphylococcus aureus
A41.1	Septicaemia due to other specified staphylococcus
A41.2	Septicaemia due to unspecified staphylococcus
A41.3	Septicaemia due to Haemophilus influenzae
A41.4	Septicaemia due to anaerobes
A41.5	Septicaemia due to other Gram-negative organisms
A41.8	Other specified septicaemia
A41.9	Septicaemia, unspecified
A42.0	Pulmonary actinomycosis
A42.1	Abdominal actinomycosis
A42.2	Cervicofacial actinomycosis
A42.7	Actinomycotic septicaemia
A42.8	Other forms of actinomycosis
A42.9	Actinomycosis, unspecified
A46	Erysipelas
A48.0	Gas gangrene
A48.8	Other specified bacterial diseases
A49.0	Staphylococcal infection, unspecified
A49.1	Streptococcal infection, unspecified
A49.2	Haemophilus influenzae infection, unspecified
A49.3	Mycoplasma infection, unspecified
A49.8	Other bacterial infections of unspecified site
A49.9	Bacterial infection, unspecified
A54.0	Gonococcal infection of lower genito-urinary tract without periurethral or accessory gland abscess
A54.2	Gonococcal pelviperitonitis and other gonococcal genito-urinary infections
A54.3	Gonococcal infection of eye
A54.4	Gonococcal infection of musculoskeletal system
A54.5	Gonococcal pharyngitis
A54.6	Gonococcal infection of anus and rectum
A54.8	Other gonococcal infections
A54.9	Gonococcal infection, unspecified
A56.4	Chlamydial infection of pharynx
A57	Chancroid
A66.0	Initial lesions of yaws
A66.1	Multiple papillomata and wet crab yaws
A66.2	Other early skin lesions of yaws
A66.3	Hyperkeratosis of yaws
A66.4	Gummata and ulcers of yaws
A66.6	Bone and joint lesions of yaws
A66.7	Other manifestations of yaws

A66.8	Latent yaws
A66.9	Yaws, unspecified
A67.0	Primary lesions of pinta
A67.1	Intermediate lesions of pinta
A67.2	Late lesions of pinta
A67.3	Mixed lesions of pinta
A67.9	Pinta, unspecified
A68.0	Louse-borne relapsing fever
A68.1	Tick-borne relapsing fever
A68.9	Relapsing fever, unspecified
A69.1	Other Vincent's infections
A70	Chlamydia psittaci infection
A71	Trachoma
A71.0	Initial stage of trachoma
A71.1	Active stage of trachoma
A71.9	Trachoma, unspecified
A74.0	Chlamydial conjunctivitis
A74.8	Other chlamydial diseases
A74.9	Chlamydial infection, unspecified
A75.0	Epidemic louse-borne typhus fever due to Rickettsia prowazekii
A75.1	Recrudescent typhus [Brill's disease]
A75.2	Typhus fever due to Rickettsia typhi
A75.3	Typhus fever due to Rickettsia tsutsugamushi
A75.9	Typhus fever, unspecified
A77.0	Spotted fever due to Rickettsia rickettsii
A79.0	Trench fever
A79.8	Other specified rickettsioses
A79.9	Rickettsiosis, unspecified
A80.9	Acute poliomyelitis, unspecified
A81.0	Creutzfeldt-Jakob disease
A81.8	Other atypical virus infections of central nervous system
A81.9	Atypical virus infection of central nervous system, unspecified
A82.0	Sylvatic rabies
A82.1	Urban rabies
A82.9	Rabies, unspecified
A83.0	Japanese encephalitis
A83.6	Rocio virus disease
A83.8	Other mosquito-borne viral encephalitis
A83.9	Mosquito-borne viral encephalitis, unspecified
A84.0	Far Eastern tick-borne encephalitis [Russian spring-summer encephalitis]
A84.8	Other tick-borne viral encephalitis
A84.9	Tick-borne viral encephalitis, unspecified
A85.0	Enteroviral encephalitis

A85.1	Adenoviral encephalitis
A85.2	Arthropod-borne viral encephalitis, unspecified
A85.8	Other specified viral encephalitis
A86	Unspecified viral encephalitis
A87.0	Enteroviral meningitis
A87.1	Adenoviral meningitis
A87.2	Lymphocytic choriomeningitis
A87.8	Other viral meningitis
A87.9	Viral meningitis, unspecified
A88.0	Enteroviral exanthematous fever [Boston exanthem]
A88.8	Other specified viral infections of central nervous system
A89	Unspecified viral infection of central nervous system
A90	Dengue fever [classical dengue]
A91	Dengue haemorrhagic fever
A92.0	Chikungunya virus disease
A92.3	West Nile fever
A92.8	Other specified mosquito-borne viral fevers
A92.9	Mosquito-borne viral fever, unspecified
A93.0	Oropouche virus disease
A93.8	Other specified arthropod-borne viral fevers
A94	Unspecified arthropod-borne viral fever
A95.0	Sylvatic yellow fever
A95.1	Urban yellow fever
A95.9	Yellow fever, unspecified
A96.0	Junin haemorrhagic fever
A96.8	Other arenaviral haemorrhagic fevers
A96.9	Arenaviral haemorrhagic fever, unspecified
A98.0	Crimean-Congo haemorrhagic fever
A98.3	Marburg virus disease
A98.4	Ebola virus disease
A98.8	Other specified viral haemorrhagic fevers
A99	Unspecified viral haemorrhagic fever
B00.0	Eczema herpeticum
B00.1	Herpesviral vesicular dermatitis
B00.2	Herpesviral gingivostomatitis and pharyngotonsillitis
B00.3	Herpesviral meningitis
B00.4	Herpesviral encephalitis
B00.5	Herpesviral ocular disease
B00.7	Disseminated herpesviral disease
B00.8	Other forms of herpesviral infection
B00.9	Herpesviral infection, unspecified
B01.0	Varicella meningitis
B01.1	Varicella encephalitis

B01.2	Varicella pneumonia
B01.8	Varicella with other complications
B01.9	Varicella without complications
B02.0	Zoster encephalitis
B02.1	Zoster meningitis
B02.2	Zoster with other nervous system involvement
B02.3	Zoster ocular disease
B02.7	Disseminated zoster
B02.8	Zoster with other complications
B02.9	Zoster without complication
B03	Smallpox
B05.0	Measles complicated by encephalitis
B05.1	Measles complicated by meningitis
B05.2	Measles complicated by pneumonia
B05.3	Measles complicated by otitis media
B05.4	Measles with intestinal complications
B05.8	Measles with other complications
B05.9	Measles without complication
B06.0	Rubella with neurological complications
B06.8	Rubella with other complications
B06.9	Rubella without complication
B07	Viral warts
B08.0	Other orthopoxvirus infections
B08.4	Enteroviral vesicular stomatitis with exanthem
B08.5	Enteroviral vesicular pharyngitis
B08.8	Other specified viral infections characterised by skin and mucous membrane lesions
B09	Unspecified viral infection characterised by skin and mucous membrane lesions
B16.0	Acute hepatitis B with delta-agent (coinfection) with hepatic coma
B16.1	Acute hepatitis B with delta-agent (coinfection) without hepatic coma
B16.2	Acute hepatitis B without delta-agent with hepatic coma
B16.9	Acute hepatitis B without delta-agent and without hepatic coma
B17.0	Acute delta-(super) infection of hepatitis B carrier
B17.1	Acute hepatitis C
B17.8	Other specified acute viral hepatitis
B17.9	Acute viral hepatitis, unspecified
B18.0	Chronic viral hepatitis B with delta-agent
B18.1	Chronic viral hepatitis B without delta-agent
B18.2	Chronic viral hepatitis C
B18.8	Other chronic viral hepatitis
B18.9	Chronic viral hepatitis, unspecified
B19.0	Unspecified viral hepatitis with coma
B19.9	Unspecified viral hepatitis without coma
B25.0	Cytomegaloviral pneumonitis

B25.1	Cytomegaloviral hepatitis
B25.2	Cytomegaloviral pancreatitis
B25.8	Other cytomegaloviral diseases
B25.9	Cytomegaloviral disease, unspecified
B26.0	Mumps orchitis
B26.1	Mumps meningitis
B26.2	Mumps encephalitis
B26.3	Mumps pancreatitis
B26.8	Mumps with other complications
B26.9	Mumps without complication
B27.0	Gammaherpesviral mononucleosis
B27.1	Cytomegaloviral mononucleosis
B27.8	Other infectious mononucleosis
B27.9	Infectious mononucleosis, unspecified
B30.0	Keratoconjunctivitis due to adenovirus
B30.1	Conjunctivitis due to adenovirus
B30.2	Viral pharyngoconjunctivitis
B30.3	Acute epidemic haemorrhagic conjunctivitis (enteroviral)
B30.8	Other viral conjunctivitis
B30.9	Viral conjunctivitis, unspecified
B33.0	Epidemic myalgia
B33.2	Viral carditis
B33.3	Retrovirus infections, not elsewhere classified
B33.4	Hantavirus (cardio-)pulmonary syndrome [HPS] [HCPS]
B33.8	Other specified viral diseases
B34.0	Adenovirus infection, unspecified
B34.1	Enterovirus infection, unspecified
B34.3	Parvovirus infection, unspecified
B34.4	Papovirus infection, unspecified
B34.8	Other viral infections of unspecified site
B34.9	Viral infection, unspecified
B35.9	Dermatophytosis, unspecified
B36.8	Other specified superficial mycoses
B37.0	Candidal stomatitis
B37.1	Pulmonary candidiasis
B37.2	Candidiasis of skin and nail
B37.3	Candidiasis of vulva and vagina
B37.4	Candidiasis of other urogenital sites
B37.5	Candidal meningitis
B37.6	Candidal endocarditis
B37.7	Candidal septicaemia
B37.8	Candidiasis of other sites
B37.9	Candidiasis, unspecified

B38.0	Acute pulmonary coccidioidomycosis
B38.1	Chronic pulmonary coccidioidomycosis
B38.2	Pulmonary coccidioidomycosis, unspecified
B38.3	Cutaneous coccidioidomycosis
B38.4	Coccidioidomycosis meningitis
B41.0	Pulmonary paracoccidioidomycosis
B41.7	Disseminated paracoccidioidomycosis
B41.8	Other forms of paracoccidioidomycosis
B41.9	Paracoccidioidomycosis, unspecified
B43.1	Phaeomycotic brain abscess
B43.2	Subcutaneous phaeomycotic abscess and cyst
B44.0	Invasive pulmonary aspergillosis
B44.1	Other pulmonary aspergillosis
B44.2	Tonsillar aspergillosis
B44.7	Disseminated aspergillosis
B44.8	Other forms of aspergillosis
B44.9	Aspergillosis, unspecified
B45.0	Pulmonary cryptococcosis
B45.1	Cerebral cryptococcosis
B45.2	Cutaneous cryptococcosis
B45.3	Osseous cryptococcosis
B45.7	Disseminated cryptococcosis
B45.8	Other forms of cryptococcosis
B45.9	Cryptococcosis, unspecified
B46.8	Other zygomycoses
B47.1	Actinomycetoma
B48.7	Opportunistic mycoses
B48.8	Other specified mycoses
B50.0	Plasmodium falciparum malaria with cerebral complications
B50.8	Other severe and complicated Plasmodium falciparum malaria
B50.9	Plasmodium falciparum malaria, unspecified
B51.0	Plasmodium vivax malaria with rupture of spleen
B51.8	Plasmodium vivax malaria with other complications
B51.9	Plasmodium vivax malaria without complication
B52.0	Plasmodium malariae malaria with nephropathy
B52.8	Plasmodium malariae malaria with other complications
B52.9	Plasmodium malariae malaria without complication
B53.0	Plasmodium ovale malaria
B53.1	Malaria due to simian plasmodia
B53.8	Other parasitologically confirmed malaria, not elsewhere classified
B54	Unspecified malaria
B55.0	Visceral leishmaniasis
B55.1	Cutaneous leishmaniasis

B55.2	Mucocutaneous leishmaniasis
B55.9	Leishmaniasis, unspecified
B56.0	Gambiense trypanosomiasis
B56.1	Rhodesiense trypanosomiasis
B56.9	African trypanosomiasis, unspecified
B58	Toxoplasmosis
B58.0	Toxoplasma oculopathy
B58.1	Toxoplasma hepatitis
B58.2	Toxoplasma meningoencephalitis
B58.3	Pulmonary toxoplasmosis
B58.8	Toxoplasmosis with other organ involvement
B58.9	Toxoplasmosis, unspecified
B60.0	Babesiosis
B60.1	Acanthamoebiasis
B60.2	Negleriasis
B60.8	Other specified protozoal diseases
B64	Unspecified protozoal disease
B65	Schistosomiasis [bilharziasis]
B65.0	Schistosomiasis due to Schistosoma haematobium [urinary schistosomiasis]
B65.1	Schistosomiasis due to Schistosoma Mansoni [intestinal schistosomiasis]
B65.2	Schistosomiasis due to Schistosoma japonicum
B65.8	Other schistosomiases
B65.9	Schistosomiasis, unspecified
B67.8	Echinococcosis, unspecified, of liver
B67.9	Echinococcosis, other and unspecified
B71.0	Hymenolepiasis
B71.8	Other specified cestode infections
B71.9	Cestode infection, unspecified
B76.1	Necatoriasis
B81.0	Anisakiasis
B81.4	Mixed intestinal helminthiases
B81.8	Other specified intestinal helminthiases
B82.0	Intestinal helminthiasis, unspecified
B82.9	Intestinal parasitism, unspecified
B83.0	Visceral larva migrans
B83.8	Other specified helminthiases
B83.9	Helminthiasis, unspecified
B85.0	Pediculosis due to Pediculus humanus capitis
B85.1	Pediculosis due to Pediculus humanus corporis
B85.2	Pediculosis, unspecified
B85.4	Mixed pediculosis and phthiriasis
B87.0	Cutaneous myiasis
B87.1	Wound myiasis

B87.2	Ocular myiasis
B87.3	Nasopharyngeal myiasis
B87.4	Aural myiasis
B87.8	Myiasis of other sites
B87.9	Myiasis, unspecified
B88.0	Other acariasis
B89	Unspecified parasitic disease
B91	Sequelae of poliomyelitis
B92	Sequelae of leprosy
B94.0	Sequelae of trachoma
B94.1	Sequelae of viral encephalitis
B94.2	Sequelae of viral hepatitis
B94.8	Sequelae of other specified infectious and parasitic diseases
B94.9	Sequelae of unspecified infectious or parasitic disease
B95.0	Streptococcus, group A, as the cause of diseases classified to other chapters
B95.1	Streptococcus, group B, as the cause of diseases classified to other chapters
B95.2	Streptococcus, group D, as the cause of diseases classified to other chapters
B95.3	Streptococcus pneumoniae as the cause of diseases classified to other chapters
B95.4	Other streptococcus as the cause of diseases classified to other chapters
B95.5	Unspecified streptococcus as the cause of diseases classified to other chapters
B95.6	Staphylococcus aureus as the cause of diseases classified to other chapters
B95.7	Other staphylococcus as the cause of diseases classified to other chapters
B95.8	Unspecified staphylococcus as the cause of diseases classified to other chapters
B96.0	Mycoplasma pneumoniae [M. pneumoniae] as the cause of diseases classified to other chapters
B96.1	Klebsiella pneumoniae [K. pneumoniae] as the cause of diseases classified to other chapters
B96.2	Escherichia coli [E. coli] as the cause of diseases classified to other chapters
B96.3	Haemophilus influenzae [H. influenzae] as the cause of diseases classified to other chapters
B96.4	Proteus (mirabilis)(morganii) as the cause of diseases classified to other chapters
B96.5	Pseudomonas (aeruginosa)(mallei)(pseudomallei) as the cause of diseases classified to other chapters
B96.7	Clostridium perfringens [C. perfringens] as the cause of diseases classified to other chapters
B96.8	Other specified bacterial agents as the cause of diseases classified to other chapters
B97.0	Adenovirus as the cause of diseases classified to other chapters
B97.1	Enterovirus as the cause of diseases classified to other chapters
B97.2	Coronavirus as the cause of diseases classified to other chapters
B97.3	Retrovirus as the cause of diseases classified to other chapters
B97.4	Respiratory synctial virus as the cause of diseases classified to other chapters
B97.5	Reovirus as the cause of diseases classified to other chapters
B97.6	Parvovirus as the cause of diseases classified to other chapters
B97.7	Papillomavirus as the cause of diseases classified to other chapters
B97.8	Other viral agents as the cause of diseases classified to other chapters
D47.5	Chronic eosinophilic leukaemia [hypereosinophilic syndrome]

D73.3	Abscess of spleen
E32.1	Abscess of thymus
G00.0	Haemophilus meningitis
G00.1	Pneumococcal meningitis
G00.2	Streptococcal meningitis
G00.3	Staphylococcal meningitis
G00.8	Other bacterial meningitis
G00.9	Bacterial meningitis, unspecified
G01	Meningitis in bacterial diseases classified elsewhere
G02.0	Meningitis in viral diseases classified elsewhere
G02.1	Meningitis in mycoses
G02.8	Meningitis in other specified infectious and parasitic diseases classified elsewhere
G03.0	Nonpyogenic meningitis
G03.1	Chronic meningitis
G03.2	Benign recurrent meningitis [Mollaret]
G03.8	Meningitis due to other specified causes
G03.9	Meningitis, unspecified
G04.2	Bacterial meningoencephalitis and meningomyelitis, not elsewhere classified
G05.0	Encephalitis, myelitis and encephalomyelitis in bacterial diseases classified elsewhere
G05.1	Encephalitis, myelitis and encephalomyelitis in viral diseases classified elsewhere
G05.2	Encephalitis, myelitis and encephalomyelitis in other infectious and parasitic diseases classified elsewhere
G06.0	Intracranial abscess and granuloma
G06.1	Intraspinal abscess and granuloma
G06.2	Extradural and subdural abscess, unspecified
G07	Intracranial and intraspinal abscess and granuloma in diseases classified elsewhere
G14	Postpolio syndrome
G53.0	Postzoster neuralgia
G53.1	Multiple cranial nerve palsies in infectious and parasitic diseases classified elsewhere
G63.0	Polyneuropathy in infectious and parasitic diseases classified elsewhere
G73.1	Eaton-Lambert syndrome
G73.4	Myopathy in infectious and parasitic diseases classified elsewhere
G93.3	Postviral fatigue syndrome
G94.0	Hydrocephalus in infectious and parasitic diseases classified elsewhere
H03.0	Parasitic infestation of eyelid in diseases classified elsewhere
H06.1	Parasitic infestation of orbit in diseases classified elsewhere
H10.0	Mucopurulent conjunctivitis
H10.2	Other acute conjunctivitis
H10.3	Acute conjunctivitis, unspecified
H10.4	Chronic conjunctivitis
H10.5	Blepharoconjunctivitis
H10.8	Other conjunctivitis
H10.9	Conjunctivitis, unspecified
H13.0	Filarial infection of conjunctiva

H13.1	Conjunctivitis in infectious and parasitic diseases classified elsewhere
H13.2	Conjunctivitis in other diseases classified elsewhere
H16.1	Other superficial keratitis without conjunctivitis
H16.2	Keratoconjunctivitis
H19.1	Herpesviral keratitis and keratoconjunctivitis
H19.2	Keratitis and keratoconjunctivitis in other infectious and parasitic diseases classified elsewhere
H19.3	Keratitis and keratoconjunctivitis in other diseases classified elsewhere
H22.0	Iridocyclitis in infectious and parasitic diseases classified elsewhere
H32.0	Chorioretinal inflammation in infectious and parasitic diseases classified elsewhere
H60.0	Abscess of external ear
H60.1	Cellulitis of external ear
H60.2	Malignant otitis externa
H60.3	Other infective otitis externa
H60.5	Acute otitis externa, non-infective
H60.8	Other otitis externa
H60.9	Otitis externa, unspecified
H62.0	Otitis externa in bacterial diseases classified elsewhere
H62.1	Otitis externa in viral diseases classified elsewhere
H62.3	Otitis externa in other infectious and parasitic diseases classified elsewhere
H62.4	Otitis externa in other diseases classified elsewhere
H65.0	Acute serous otitis media
H65.1	Other acute nonsuppurative otitis media
H65.2	Chronic serous otitis media
H65.3	Chronic mucoid otitis media
H65.4	Other chronic nonsuppurative otitis media
H65.9	Nonsuppurative otitis media, unspecified
H66.0	Acute suppurative otitis media
H66.1	Chronic tubotympanic suppurative otitis media
H66.2	Chronic atticoantral suppurative otitis media
H66.3	Other chronic suppurative otitis media
H66.4	Suppurative otitis media, unspecified
H66.9	Otitis media, unspecified
H67.0	Otitis media in bacterial diseases classified elsewhere
H67.1	Otitis media in viral diseases classified elsewhere
H67.8	Otitis media in other diseases classified elsewhere
H70.0	Acute mastoiditis
H70.1	Chronic mastoiditis
H70.8	Other mastoiditis and related conditions
H70.9	Mastoiditis, unspecified
H73.0	Acute myringitis
H73.1	Chronic myringitis
H75.0	Mastoiditis in infectious and parasitic diseases classified elsewhere
H94.0	Acoustic neuritis in infectious and parasitic diseases classified elsewhere

I32.0	Pericarditis in bacterial diseases classified elsewhere
I32.1	Pericarditis in other infectious and parasitic diseases classified elsewhere
I33.0	Acute and subacute infective endocarditis
I33.9	Acute endocarditis, unspecified
I38	Endocarditis, valve unspecified
I39	Endocarditis and heart valve disorders in diseases classified elsewhere
I39.1	Aortic valve disorders in diseases classified elsewhere
I39.2	Tricuspid valve disorders in diseases classified elsewhere
I39.3	Pulmonary valve disorders in diseases classified elsewhere
I39.4	Multiple valve disorders in diseases classified elsewhere
I39.8	Endocarditis, valve unspecified, in diseases classified elsewhere
I40.0	Infective myocarditis
I41.0	Myocarditis in bacterial diseases classified elsewhere
I41.1	Myocarditis in viral diseases classified elsewhere
I41.2	Myocarditis in other infectious and parasitic diseases classified elsewhere
I42.3	Endomyocardial (eosinophilic) disease
I43.0	Cardiomyopathy in infectious and parasitic diseases classified elsewhere
I52.0	Other heart disorders in bacterial diseases classified elsewhere
I52.1	Other heart disorders in other infectious and parasitic diseases classified elsewhere
I68.1	Cerebral arteritis in infectious and parasitic diseases classified elsewhere
I98.1	Cardiovascular disorders in other infectious and parasitic diseases classified elsewhere
J00	Acute nasopharyngitis [common cold]
J01.0	Acute maxillary sinusitis
J01.1	Acute frontal sinusitis
J01.2	Acute ethmoidal sinusitis
J01.3	Acute sphenoidal sinusitis
J01.4	Acute pansinusitis
J01.8	Other acute sinusitis
J01.9	Acute sinusitis, unspecified
J02.0	Streptococcal pharyngitis
J02.8	Acute pharyngitis due to other specified organisms
J02.9	Acute pharyngitis, unspecified
J03.0	Streptococcal tonsillitis
J03.8	Acute tonsillitis due to other specified organisms
J03.9	Acute tonsillitis, unspecified
J06.0	Acute laryngopharyngitis
J06.8	Other acute upper respiratory infections of multiple sites
J06.9	Acute upper respiratory infection, unspecified
J09	Influenza due to certain identified influenza virus
J10.0	Influenza with pneumonia, influenza virus identified
J10.1	Influenza with other respiratory manifestations, influenza virus identified
J10.8	Influenza with other manifestations, influenza virus identified
J11.0	Influenza with pneumonia, virus not identified

J11.1	Influenza with other respiratory manifestations, virus not identified
J11.8	Influenza with other manifestations, virus not identified
J12	Viral pneumonia, not elsewhere classified
J12.0	Adenoviral pneumonia
J12.1	Respiratory syncytial virus pneumonia
J12.2	Parainfluenza virus pneumonia
J12.3	Human metapneumovirus pneumonia
J12.8	Other viral pneumonia
J12.9	Viral pneumonia, unspecified
J13	Pneumonia due to Streptococcus pneumoniae
J14	Pneumonia due to Haemophilus influenzae
J15.0	Pneumonia due to Klebsiella pneumoniae
J15.1	Pneumonia due to Pseudomonas
J15.2	Pneumonia due to staphylococcus
J15.3	Pneumonia due to streptococcus, group B
J15.4	Pneumonia due to other streptococci
J15.5	Pneumonia due to Escherichia coli
J15.6	Pneumonia due to other aerobic Gram-negative bacteria
J15.7	Pneumonia due to Mycoplasma pneumoniae
J15.8	Other bacterial pneumonia
J15.9	Bacterial pneumonia, unspecified
J16.0	Chlamydial pneumonia
J16.8	Pneumonia due to other specified infectious organisms
J17.0	Pneumonia in bacterial diseases classified elsewhere
J17.1	Pneumonia in viral diseases classified elsewhere
J17.2	Pneumonia in mycoses
J17.3	Pneumonia in parasitic diseases
J17.8	Pneumonia in other diseases classified elsewhere
J18.0	Bronchopneumonia, unspecified
J18.1	Lobar pneumonia, unspecified
J18.2	Hypostatic pneumonia, unspecified
J18.8	Other pneumonia, organism unspecified
J18.9	Pneumonia, unspecified
J20.0	Acute bronchitis due to Mycoplasma pneumoniae
J20.1	Acute bronchitis due to Haemophilus influenzae
J20.2	Acute bronchitis due to streptococcus
J20.3	Acute bronchitis due to coxsackie virus
J20.4	Acute bronchitis due to parainfluenza virus
J20.5	Acute bronchitis due to respiratory syncytial virus
J20.6	Acute bronchitis due to rhinovirus
J20.7	Acute bronchitis due to echovirus
J21.0	Acute bronchiolitis due to respiratory syncytial virus
J21.1	Acute bronchiolitis due to human metapneumovirus

J22	Unspecified acute lower respiratory infection
J31	Chronic rhinitis, nasopharyngitis and pharyngitis
J31.0	Chronic rhinitis
J31.1	Chronic nasopharyngitis
J31.2	Chronic pharyngitis
J32.0	Chronic maxillary sinusitis
J32.1	Chronic frontal sinusitis
J32.2	Chronic ethmoidal sinusitis
J32.3	Chronic sphenoidal sinusitis
J32.4	Chronic pansinusitis
J32.8	Other chronic sinusitis
J32.9	Chronic sinusitis, unspecified
J34.0	Abscess, furuncle and carbuncle of nose
J35.0	Chronic tonsillitis
J36	Peritonsillar abscess
J39.0	Retropharyngeal and parapharyngeal abscess
J39.1	Other abscess of pharynx
J44.0	Chronic obstructive pulmonary disease with acute lower respiratory infection
J65	Pneumoconiosis associated with tuberculosis
J85.0	Gangrene and necrosis of lung
J85.1	Abscess of lung with pneumonia
J85.2	Abscess of lung without pneumonia
J85.3	Abscess of mediastinum
K04.6	Periapical abscess with sinus
K04.7	Periapical abscess without sinus
K11.3	Abscess of salivary gland
K12.2	Cellulitis and abscess of mouth
K23.0	Tuberculous oesophagitis
K35.2	Acute appendicitis with generalized peritonitis
K35.3	Acute appendicitis with localized peritonitis
K35.8	Acute appendicitis, other and unspecified
K36	Other appendicitis
K37	Unspecified appendicitis
K51.5	Mucosal proctocolitis
K52.3	Indeterminate colitis
K57.0	Diverticular disease of small intestine with perforation and abscess
K57.1	Diverticular disease of small intestine without perforation or abscess
K57.2	Diverticular disease of large intestine with perforation and abscess
K57.3	Diverticular disease of large intestine without perforation or abscess
K57.4	Diverticular disease of both small and large intestine with perforation and abscess
K57.5	Diverticular disease of both small and large intestine without perforation or abscess
K57.8	Diverticular disease of intestine, part unspecified, with perforation and abscess
K57.9	Diverticular disease of intestine, part unspecified, without perforation or abscess

K61.0	Anal abscess
K61.1	Rectal abscess
K61.2	Anorectal abscess
K61.3	Ischiorectal abscess
K61.4	Intrasphincteric abscess
K63.0	Abscess of intestine
K65.0	Acute peritonitis
K65.8	Other peritonitis
K65.9	Peritonitis, unspecified
K67.0	Chlamydial peritonitis
K67.1	Gonococcal peritonitis
K67.2	Syphilitic peritonitis
K67.3	Tuberculous peritonitis
K75.0	Abscess of liver
K77.0	Liver disorders in infectious and parasitic diseases classified elsewhere
K80.0	Calculus of gallbladder with acute cholecystitis
K80.1	Calculus of gallbladder with other cholecystitis
K80.2	Calculus of gallbladder without cholecystitis
K80.3	Calculus of bile duct with cholangitis
K80.36	Calculus of bile duct with acute and chronic cholangitis without obstruction
K80.4	Calculus of bile duct with cholecystitis
K80.5	Calculus of bile duct without cholangitis or cholecystitis
K81.0	Acute cholecystitis
K81.1	Chronic cholecystitis
K81.9	Cholecystitis, unspecified
K83.0	Cholangitis
K93.0	Tuberculous disorders of intestines, peritoneum and mesenteric glands
L00	Staphylococcal scalded skin syndrome
L02.0	Cutaneous abscess, furuncle and carbuncle of face
L02.1	Cutaneous abscess, furuncle and carbuncle of neck
L02.2	Cutaneous abscess, furuncle and carbuncle of trunk
L02.3	Cutaneous abscess, furuncle and carbuncle of buttock
L02.4	Cutaneous abscess, furuncle and carbuncle of limb
L02.8	Cutaneous abscess, furuncle and carbuncle of other sites
L02.9	Cutaneous abscess, furuncle and carbuncle, unspecified
L03	Cellulitis
L03.0	Cellulitis of finger and toe
L03.1	Cellulitis of other parts of limb
L03.2	Cellulitis of face
L03.3	Cellulitis of trunk
L03.8	Cellulitis of other sites
L03.9	Cellulitis, unspecified
L05.0	Pilonidal cyst with abscess

L05.9	Pilonidal cyst without abscess
L92.2	Granuloma faciale [eosinophilic granuloma of skin]
L98.3	Eosinophilic cellulitis [Wells]
M00	Pyogenic arthritis
M00.0	Staphylococcal arthritis and polyarthritis
M00.00	Staphylococcal arthritis and polyarthritis (Multiple sites)
M00.01	Staphylococcal arthritis and polyarthritis (Shoulder region)
M00.02	Staphylococcal arthritis and polyarthritis (Upper arm)
M00.03	Staphylococcal arthritis and polyarthritis (Forearm)
M00.04	Staphylococcal arthritis and polyarthritis (Hand)
M00.05	Staphylococcal arthritis and polyarthritis (Pelvic region and thigh)
M00.06	Staphylococcal arthritis and polyarthritis (Lower leg)
M00.07	Staphylococcal arthritis and polyarthritis (Ankle and foot)
M00.08	Staphylococcal arthritis and polyarthritis (Other)
M00.09	Staphylococcal arthritis and polyarthritis (Site unspecified)
M00.1	Pneumococcal arthritis and polyarthritis
M00.10	Pneumococcal arthritis and polyarthritis (Multiple sites)
M00.11	Pneumococcal arthritis and polyarthritis (Shoulder region)
M00.12	Pneumococcal arthritis and polyarthritis (Upper arm)
M00.13	Pneumococcal arthritis and polyarthritis (Forearm)
M00.14	Pneumococcal arthritis and polyarthritis (Hand)
M00.15	Pneumococcal arthritis and polyarthritis (Pelvic region and thigh)
M00.16	Pneumococcal arthritis and polyarthritis (Lower leg)
M00.17	Pneumococcal arthritis and polyarthritis (Ankle and foot)
M00.18	Pneumococcal arthritis and polyarthritis (Other)
M00.19	Pneumococcal arthritis and polyarthritis (Site unspecified)
M00.2	Other streptococcal arthritis and polyarthritis
M00.20	Other streptococcal arthritis and polyarthritis (Multiple sites)
M00.21	Other streptococcal arthritis and polyarthritis (Shoulder region)
M00.22	Other streptococcal arthritis and polyarthritis (Upper arm)
M00.23	Other streptococcal arthritis and polyarthritis (Forearm)
M00.24	Other streptococcal arthritis and polyarthritis (Hand)
M00.25	Other streptococcal arthritis and polyarthritis (Pelvic region and thigh)
M00.26	Other streptococcal arthritis and polyarthritis (Lower leg)
M00.27	Other streptococcal arthritis and polyarthritis (Ankle and foot)
M00.28	Other streptococcal arthritis and polyarthritis (Other)
M00.29	Other streptococcal arthritis and polyarthritis (Site unspecified)
M00.8	Arthritis and polyarthritis due to other specified bacterial agents
M00.80	Arthritis and polyarthritis due to other specified bacterial agents (Multiple sites)
M00.81	Arthritis and polyarthritis due to other specified bacterial agents (Shoulder region)
M00.82	Arthritis and polyarthritis due to other specified bacterial agents (Upper arm)
M00.83	Arthritis and polyarthritis due to other specified bacterial agents (Forearm)
M00.84	Arthritis and polyarthritis due to other specified bacterial agents (Hand)

M00.85	Arthritis and polyarthritis due to other specified bacterial agents (Pelvic region and thigh)
M00.86	Arthritis and polyarthritis due to other specified bacterial agents (Lower leg)
M00.87	Arthritis and polyarthritis due to other specified bacterial agents (Ankle and foot)
M00.88	Arthritis and polyarthritis due to other specified bacterial agents (Other)
M00.89	Arthritis and polyarthritis due to other specified bacterial agents (Site unspecified)
M01.0	Meningococcal arthritis
M01.00	Meningococcal arthritis (Multiple sites)
M01.01	Meningococcal arthritis (Shoulder region)
M01.02	Meningococcal arthritis (Upper arm)
M01.03	Meningococcal arthritis (Forearm)
M01.04	Meningococcal arthritis (Hand)
M01.05	Meningococcal arthritis (Pelvic region and thigh)
M01.06	Meningococcal arthritis (Lower leg)
M01.07	Meningococcal arthritis (Ankle and foot)
M01.08	Meningococcal arthritis (Other)
M01.09	Meningococcal arthritis (Site unspecified)
M01.1	Tuberculous arthritis
M01.10	Tuberculous arthritis (Multiple sites)
M01.11	Tuberculous arthritis (Shoulder region)
M01.12	Tuberculous arthritis (Upper arm)
M01.13	Tuberculous arthritis (Forearm)
M01.14	Tuberculous arthritis (Hand)
M01.15	Tuberculous arthritis (Pelvic region and thigh)
M01.16	Tuberculous arthritis (Lower leg)
M01.17	Tuberculous arthritis (Ankle and foot)
M01.18	Tuberculous arthritis (Other)
M01.19	Tuberculous arthritis (Site unspecified)
M01.3	Arthritis in other bacterial diseases classified elsewhere
M01.30	Arthritis in other bacterial diseases classified elsewhere (Multiple sites)
M01.31	Arthritis in other bacterial diseases classified elsewhere (Shoulder region)
M01.32	Arthritis in other bacterial diseases classified elsewhere (Upper arm)
M01.33	Arthritis in other bacterial diseases classified elsewhere (Forearm)
M01.34	Arthritis in other bacterial diseases classified elsewhere (Hand)
M01.35	Arthritis in other bacterial diseases classified elsewhere (Pelvic region and thigh)
M01.36	Arthritis in other bacterial diseases classified elsewhere (Lower leg)
M01.37	Arthritis in other bacterial diseases classified elsewhere (Ankle and foot)
M01.38	Arthritis in other bacterial diseases classified elsewhere (Other)
M01.39	Arthritis in other bacterial diseases classified elsewhere (Site unspecified)
M01.4	Rubella arthritis
M01.40	Rubella arthritis (Multiple sites)
M01.41	Rubella arthritis (Shoulder region)
M01.42	Rubella arthritis (Upper arm)
M01.43	Rubella arthritis (Forearm)

M01.44	Rubella arthritis (Hand)
M01.45	Rubella arthritis (Pelvic region and thigh)
M01.46	Rubella arthritis (Lower leg)
M01.47	Rubella arthritis (Ankle and foot)
M01.48	Rubella arthritis (Other)
M01.49	Rubella arthritis (Site unspecified)
M01.5	Arthritis in other viral diseases classified elsewhere
M01.50	Arthritis in other viral diseases classified elsewhere (Multiple sites)
M01.51	Arthritis in other viral diseases classified elsewhere (Shoulder region)
M01.52	Arthritis in other viral diseases classified elsewhere (Upper arm)
M01.53	Arthritis in other viral diseases classified elsewhere (Forearm)
M01.54	Arthritis in other viral diseases classified elsewhere (Hand)
M01.55	Arthritis in other viral diseases classified elsewhere (Pelvic region and thigh)
M01.56	Arthritis in other viral diseases classified elsewhere (Lower leg)
M01.57	Arthritis in other viral diseases classified elsewhere (Ankle and foot)
M01.58	Arthritis in other viral diseases classified elsewhere (Other)
M01.59	Arthritis in other viral diseases classified elsewhere (Site unspecified)
M01.6	Arthritis in mycoses
M01.60	Arthritis in mycoses (Multiple sites)
M01.61	Arthritis in mycoses (Shoulder region)
M01.62	Arthritis in mycoses (Upper arm)
M01.63	Arthritis in mycoses (Forearm)
M01.64	Arthritis in mycoses (Hand)
M01.65	Arthritis in mycoses (Pelvic region and thigh)
M01.66	Arthritis in mycoses (Lower leg)
M01.67	Arthritis in mycoses (Ankle and foot)
M01.68	Arthritis in mycoses (Other)
M01.69	Arthritis in mycoses (Site unspecified)
M02.30	Reiter's disease (Multiple sites)
M02.31	Reiter's disease (Shoulder region)
M02.32	Reiter's disease (Upper arm)
M02.33	Reiter's disease (Forearm)
M02.34	Reiter's disease (Hand)
M02.35	Reiter's disease (Pelvic region and thigh)
M02.36	Reiter's disease (Lower leg)
M02.37	Reiter's disease (Ankle and foot)
M02.38	Reiter's disease (Other)
M02.39	Reiter's disease (Site unspecified)
M03.0	Postmeningococcal arthritis
M03.00	Postmeningococcal arthritis (Multiple sites)
M03.01	Postmeningococcal arthritis (Shoulder region)
M03.02	Postmeningococcal arthritis (Upper arm)
M03.03	Postmeningococcal arthritis (Forearm)

M03.04	Postmeningococcal arthritis (Hand)
M03.05	Postmeningococcal arthritis (Pelvic region and thigh)
M03.06	Postmeningococcal arthritis (Lower leg)
M03.07	Postmeningococcal arthritis (Ankle and foot)
M03.08	Postmeningococcal arthritis (Other)
M03.09	Postmeningococcal arthritis (Site unspecified)
M03.1	Postinfective arthropathy in syphilis
M03.10	Postinfective arthropathy in syphilis (Multiple sites)
M03.11	Postinfective arthropathy in syphilis (Shoulder region)
M03.12	Postinfective arthropathy in syphilis (Upper arm)
M03.13	Postinfective arthropathy in syphilis (Forearm)
M03.14	Postinfective arthropathy in syphilis (Hand)
M03.15	Postinfective arthropathy in syphilis (Pelvic region and thigh)
M03.16	Postinfective arthropathy in syphilis (Lower leg)
M03.17	Postinfective arthropathy in syphilis (Ankle and foot)
M03.18	Postinfective arthropathy in syphilis (Other)
M03.19	Postinfective arthropathy in syphilis (Site unspecified)
M35.4	Diffuse (eosinophilic) fasciitis
M46.2	Osteomyelitis of vertebra
M46.20	Osteomyelitis of vertebra (Multiple sites in spine)
M46.21	Osteomyelitis of vertebra (Occipito-atlanto-axial region)
M46.22	Osteomyelitis of vertebra (Cervical region)
M46.23	Osteomyelitis of vertebra (Cervicothoracic region)
M46.24	Osteomyelitis of vertebra (Thoracic region)
M46.25	Osteomyelitis of vertebra (Thoracolumbar region)
M46.26	Osteomyelitis of vertebra (Lumbar region)
M46.27	Osteomyelitis of vertebra (Lumbosacral region)
M46.28	Osteomyelitis of vertebra (Sacral and sacrococcygeal region)
M46.29	Osteomyelitis of vertebra (Site unspecified)
M49.0	Tuberculosis of spine
M49.00	Tuberculosis of spine (Multiple sites in spine)
M49.01	Tuberculosis of spine (Occipito-atlanto-axial region)
M49.02	Tuberculosis of spine (Cervical region)
M49.03	Tuberculosis of spine (Cervicothoracic region)
M49.04	Tuberculosis of spine (Thoracic region)
M49.05	Tuberculosis of spine (Thoracolumbar region)
M49.06	Tuberculosis of spine (Lumbar region)
M49.07	Tuberculosis of spine (Lumbosacral region)
M49.08	Tuberculosis of spine (Sacral and sacrococcygeal region)
M49.09	Tuberculosis of spine (Site unspecified)
M49.1	Brucella spondylitis
M49.10	Brucella spondylitis (Multiple sites in spine)
M49.11	Brucella spondylitis (Occipito-atlanto-axial region)

M49.12	Brucella spondylitis (Cervical region)
M49.13	Brucella spondylitis (Cervicothoracic region)
M49.14	Brucella spondylitis (Thoracic region)
M49.15	Brucella spondylitis (Thoracolumbar region)
M49.16	Brucella spondylitis (Lumbar region)
M49.17	Brucella spondylitis (Lumbosacral region)
M49.18	Brucella spondylitis (Sacral and sacrococcygeal region)
M49.19	Brucella spondylitis (Site unspecified)
M49.2	Enterobacterial spondylitis
M49.20	Enterobacterial spondylitis (Multiple sites in spine)
M49.21	Enterobacterial spondylitis (Occipito-atlanto-axial region)
M49.22	Enterobacterial spondylitis (Cervical region)
M49.23	Enterobacterial spondylitis (Cervicothoracic region)
M49.24	Enterobacterial spondylitis (Thoracic region)
M49.25	Enterobacterial spondylitis (Thoracolumbar region)
M49.26	Enterobacterial spondylitis (Lumbar region)
M49.27	Enterobacterial spondylitis (Lumbosacral region)
M49.28	Enterobacterial spondylitis (Sacral and sacrococcygeal region)
M49.29	Enterobacterial spondylitis (Site unspecified)
M49.3	Spondylopathy in other infectious and parasitic diseases classified elsewhere
M49.30	Spondylopathy in other infectious and parasitic diseases classified elsewhere (Multiple sites in spine)
M49.31	Spondylopathy in other infectious and parasitic diseases classified elsewhere (Occipito-atlanto-axial region)
M49.32	Spondylopathy in other infectious and parasitic diseases classified elsewhere (Cervical region)
M49.33	Spondylopathy in other infectious and parasitic diseases classified elsewhere (Cervicothoracic region)
M49.34	Spondylopathy in other infectious and parasitic diseases classified elsewhere (Thoracic region)
M49.35	Spondylopathy in other infectious and parasitic diseases classified elsewhere (Thoracolumbar region)
M49.36	Spondylopathy in other infectious and parasitic diseases classified elsewhere (Lumbar region)
M49.37	Spondylopathy in other infectious and parasitic diseases classified elsewhere (Lumbosacral region)
M49.38	Spondylopathy in other infectious and parasitic diseases classified elsewhere (Sacral and sacrococcygeal region)
M49.39	Spondylopathy in other infectious and parasitic diseases classified elsewhere (Site unspecified)
M63.0	Myositis in bacterial diseases classified elsewhere
M63.1	Myositis in protozoal and parasitic infections classified elsewhere
M65.0	Abscess of tendon sheath
M65.00	Abscess of tendon sheath (Multiple sites)
M65.01	Abscess of tendon sheath (Shoulder region)
M65.02	Abscess of tendon sheath (Upper arm)
M65.03	Abscess of tendon sheath (Forearm)
M65.04	Abscess of tendon sheath (Hand)
M65.05	Abscess of tendon sheath (Pelvic region and thigh)

M65.06	Abscess of tendon sheath (Lower leg)
M65.07	Abscess of tendon sheath (Ankle and foot)
M65.08	Abscess of tendon sheath (Other)
M65.09	Abscess of tendon sheath (Site unspecified)
M68.0	Synovitis and tenosynovitis in bacterial diseases classified elsewhere
M71.0	Abscess of bursa
M71.00	Abscess of bursa (Multiple sites)
M71.01	Abscess of bursa (Shoulder region)
M71.02	Abscess of bursa (Upper arm)
M71.03	Abscess of bursa (Forearm)
M71.04	Abscess of bursa (Hand)
M71.05	Abscess of bursa (Pelvic region and thigh)
M71.06	Abscess of bursa (Lower leg)
M71.07	Abscess of bursa (Ankle and foot)
M71.08	Abscess of bursa (Other)
M71.09	Abscess of bursa (Site unspecified)
M73.0	Gonococcal bursitis
M73.00	Gonococcal bursitis (Multiple sites)
M73.01	Gonococcal bursitis (Shoulder region)
M73.02	Gonococcal bursitis (Upper arm)
M73.03	Gonococcal bursitis (Forearm)
M73.04	Gonococcal bursitis (Hand)
M73.05	Gonococcal bursitis (Pelvic region and thigh)
M73.06	Gonococcal bursitis (Lower leg)
M73.07	Gonococcal bursitis (Ankle and foot)
M73.08	Gonococcal bursitis (Other)
M73.09	Gonococcal bursitis (Site unspecified)
M86.0	Acute haematogenous osteomyelitis
M86.00	Acute haematogenous osteomyelitis (Multiple sites)
M86.01	Acute haematogenous osteomyelitis (Shoulder region)
M86.02	Acute haematogenous osteomyelitis (Upper arm)
M86.03	Acute haematogenous osteomyelitis (Forearm)
M86.04	Acute haematogenous osteomyelitis (Hand)
M86.05	Acute haematogenous osteomyelitis (Pelvic region and thigh)
M86.06	Acute haematogenous osteomyelitis (Lower leg)
M86.07	Acute haematogenous osteomyelitis (Ankle and foot)
M86.08	Acute haematogenous osteomyelitis (Other)
M86.09	Acute haematogenous osteomyelitis (Site unspecified)
M86.1	Other acute osteomyelitis
M86.10	Other acute osteomyelitis (Multiple sites)
M86.11	Other acute osteomyelitis (Shoulder region)
M86.12	Other acute osteomyelitis (Upper arm)
M86.13	Other acute osteomyelitis (Forearm)

M86.14	Other acute osteomyelitis (Hand)
M86.15	Other acute osteomyelitis (Pelvic region and thigh)
M86.16	Other acute osteomyelitis (Lower leg)
M86.17	Other acute osteomyelitis (Ankle and foot)
M86.18	Other acute osteomyelitis (Other)
M86.19	Other acute osteomyelitis (Site unspecified)
M86.2	Subacute osteomyelitis
M86.20	Subacute osteomyelitis (Multiple sites)
M86.21	Subacute osteomyelitis (Shoulder region)
M86.22	Subacute osteomyelitis (Upper arm)
M86.23	Subacute osteomyelitis (Forearm)
M86.24	Subacute osteomyelitis (Hand)
M86.25	Subacute osteomyelitis (Pelvic region and thigh)
M86.26	Subacute osteomyelitis (Lower leg)
M86.27	Subacute osteomyelitis (Ankle and foot)
M86.28	Subacute osteomyelitis (Other)
M86.29	Subacute osteomyelitis (Site unspecified)
M86.3	Chronic multifocal osteomyelitis
M86.30	Chronic multifocal osteomyelitis (Multiple sites)
M86.31	Chronic multifocal osteomyelitis (Shoulder region)
M86.32	Chronic multifocal osteomyelitis (Upper arm)
M86.33	Chronic multifocal osteomyelitis (Forearm)
M86.34	Chronic multifocal osteomyelitis (Hand)
M86.35	Chronic multifocal osteomyelitis (Pelvic region and thigh)
M86.36	Chronic multifocal osteomyelitis (Lower leg)
M86.37	Chronic multifocal osteomyelitis (Ankle and foot)
M86.38	Chronic multifocal osteomyelitis (Other)
M86.39	Chronic multifocal osteomyelitis (Site unspecified)
M86.4	Chronic osteomyelitis with draining sinus
M86.40	Chronic osteomyelitis with draining sinus (Multiple sites)
M86.41	Chronic osteomyelitis with draining sinus (Shoulder region)
M86.42	Chronic osteomyelitis with draining sinus (Upper arm)
M86.43	Chronic osteomyelitis with draining sinus (Forearm)
M86.44	Chronic osteomyelitis with draining sinus (Hand)
M86.45	Chronic osteomyelitis with draining sinus (Pelvic region and thigh)
M86.46	Chronic osteomyelitis with draining sinus (Lower leg)
M86.47	Chronic osteomyelitis with draining sinus (Ankle and foot)
M86.48	Chronic osteomyelitis with draining sinus (Other)
M86.49	Chronic osteomyelitis with draining sinus (Site unspecified)
M86.5	Other chronic haematogenous osteomyelitis
M86.50	Other chronic haematogenous osteomyelitis (Multiple sites)
M86.51	Other chronic haematogenous osteomyelitis (Shoulder region)
M86.52	Other chronic haematogenous osteomyelitis (Upper arm)

M86.53	Other chronic haematogenous osteomyelitis (Forearm)
M86.54	Other chronic haematogenous osteomyelitis (Hand)
M86.55	Other chronic haematogenous osteomyelitis (Pelvic region and thigh)
M86.56	Other chronic haematogenous osteomyelitis (Lower leg)
M86.57	Other chronic haematogenous osteomyelitis (Ankle and foot)
M86.58	Other chronic haematogenous osteomyelitis (Other)
M86.59	Other chronic haematogenous osteomyelitis (Site unspecified)
M86.6	Other chronic osteomyelitis
M86.60	Other chronic osteomyelitis (Multiple sites)
M86.61	Other chronic osteomyelitis (Shoulder region)
M86.62	Other chronic osteomyelitis (Upper arm)
M86.63	Other chronic osteomyelitis (Forearm)
M86.64	Other chronic osteomyelitis (Hand)
M86.65	Other chronic osteomyelitis (Pelvic region and thigh)
M86.66	Other chronic osteomyelitis (Lower leg)
M86.67	Other chronic osteomyelitis (Ankle and foot)
M86.68	Other chronic osteomyelitis (Other)
M89.6	Osteopathy after poliomyelitis
M89.60	Osteopathy after poliomyelitis (Multiple sites)
M89.61	Osteopathy after poliomyelitis (Shoulder region)
M89.62	Osteopathy after poliomyelitis (Upper arm)
M89.63	Osteopathy after poliomyelitis (Forearm)
M89.64	Osteopathy after poliomyelitis (Hand)
M89.65	Osteopathy after poliomyelitis (Pelvic region and thigh)
M89.66	Osteopathy after poliomyelitis (Lower leg)
M89.67	Osteopathy after poliomyelitis (Ankle and foot)
M89.68	Osteopathy after poliomyelitis (Other)
M89.69	Osteopathy after poliomyelitis (Site unspecified)
N08.0	Glomerular disorders in infectious and parasitic diseases classified elsewhere
N15.1	Renal and perinephric abscess
N16.0	Renal tubulo-interstitial disorders in infectious and parasitic diseases classified elsewhere
N22.0	Calculus of urinary tract in schistosomiasis [bilharziasis]
N29.1	Other disorders of kidney and ureter in infectious and parasitic diseases classified elsewhere
N33.0	Tuberculous cystitis
N34.0	Urethral abscess
N35.1	Postinfective urethral stricture, not elsewhere classified
N39.0	Urinary tract infection, site not specified
N41.2	Abscess of prostate
N45	Orchitis and epididymitis
N45.0	Orchitis, epididymitis and epididymo-orchitis with abscess
N45.9	Orchitis, epididymitis and epididymo-orchitis without abscess
N73.0	Acute parametritis and pelvic cellulitis
N73.1	Chronic parametritis and pelvic cellulitis

N73.2	Unspecified parametritis and pelvic cellulitis
N73.3	Female acute pelvic peritonitis
N73.4	Female chronic pelvic peritonitis
N73.5	Female pelvic peritonitis, unspecified
N74.0	Tuberculous infection of cervix uteri
N74.1	Female tuberculous pelvic inflammatory disease
N75.1	Abscess of Bartholin's gland
N76.4	Abscess of vulva
N77.0	Ulceration of vulva in infectious and parasitic diseases classified elsewhere
N77.1	Vaginitis, vulvitis and vulvovaginitis in infectious and parasitic diseases classified elsewhere
O26.4	Herpes gestationis
O35.3	Maternal care for (suspected) damage to foetus from viral disease in mother
O75.3	Other infection during labour
O98.0	Tuberculosis complicating pregnancy, childbirth and the puerperium
O98.4	Viral hepatitis complicating pregnancy, childbirth and the puerperium
O98.5	Other viral diseases complicating pregnancy, childbirth and the puerperium
O98.6	Protozoal diseases complicating pregnancy, childbirth and the puerperium
O98.8	Other maternal infectious and parasitic diseases complicating pregnancy, childbirth and the puerperium
O98.9	Unspecified maternal infectious or parasitic disease complicating pregnancy, childbirth and the puerperium
R57.2	Septic shock
